# Novel Epigenetic Eight-Gene Signature Predictive of Poor Prognosis and MSI-Like Phenotype in Human Metastatic Colorectal Carcinomas

**DOI:** 10.3390/cancers13010158

**Published:** 2021-01-05

**Authors:** Valentina Condelli, Giovanni Calice, Alessandra Cassano, Michele Basso, Maria Grazia Rodriquenz, Angela Zupa, Francesca Maddalena, Fabiana Crispo, Michele Pietrafesa, Michele Aieta, Alessandro Sgambato, Giampaolo Tortora, Pietro Zoppoli, Matteo Landriscina

**Affiliations:** 1 Laboratory of Preclinical and Translational Research, Istituto di Ricovero e Cura a Carattere Scientifico Centro di Riferimento Oncologico della Basilicata (IRCCS-CROB), 85028 Potenza, Italy; valentina.condelli@crob.it (V.C.); giovanni.calice@crob.it (G.C.); francescamaddalena77@gmail.com (F.M.); fabiana.crispo@crob.it (F.C.); michele.pietrafesa@crob.it (M.P.); alessandro.sgambato@crob.it (A.S.); 2 Medical Oncology Unit, Policlinico Universitario Agostino Gemelli IRCCS, 00168 Rome, Italy; alessandra.cassano@policlinicogemelli.it (A.C.); michele.basso@policlinicogemelli.it (M.B.); giampaolo.tortora@policlinicogemelli.it (G.T.); 3 Medical Oncology Unit, Istituto di Ricovero e Cura a Carattere Scientifico Centro di Riferimento Oncologico della Basilicata (IRCCS-CROB), 85028 Potenza, Italy; mg.rodriquenz@operapadrepio.it (M.G.R.); michele.aieta@crob.it (M.A.); 4 Pathology Unit, Istituto di Ricovero e Cura a Carattere Scientifico Centro di Riferimento Oncologico della Basilicata (IRCCS-CROB), 85028 Potenza, Italy; angela.zupa@crob.it; 5 Medical Oncology Unit, Department of Medical and Surgical Sciences, University of Foggia, 71122 Foggia, Italy

**Keywords:** promoter methylation, colorectal carcinoma, gene signature, prognosis, MSI-like signature, CIMP status

## Abstract

**Simple Summary:**

The global methylation profile of two human metastatic colorectal carcinoma subgroups with significantly different outcomes (primary-resistant versus drug-sensitive tumors) was analyzed and compared with the gene expression and methylation data from The Cancer Genome Atlas COlon ADenocarcinoma (TCGA COAD) metastatic colorectal carcinoma dataset with the aim to identify a prognostic signature of functionally methylated genes. A novel epigenetic eight-gene signature, with hypermethylation of the promoter regions, was identified and validated for its capacity to predict poor outcome, which had a CpG-island methylator phenotype (CIMP)-high status and microsatellite instability (MSI)-like phenotype.

**Abstract:**

Epigenetics is involved in tumor progression and drug resistance in human colorectal carcinoma (CRC). This study addressed the hypothesis that the DNA methylation profiling may predict the clinical behavior of metastatic CRCs (mCRCs). The global methylation profile of two human mCRC subgroups with significantly different outcome was analyzed and compared with gene expression and methylation data from The Cancer Genome Atlas COlon ADenocarcinoma (TCGA COAD) and the NCBI GENE expression Omnibus repository (GEO) GSE48684 mCRCs datasets to identify a prognostic signature of functionally methylated genes. A novel epigenetic signature of eight hypermethylated genes was characterized that was able to identify mCRCs with poor prognosis, which had a CpG-island methylator phenotype (CIMP)-high and microsatellite instability (MSI)-like phenotype. Interestingly, methylation events were enriched in genes located on the q-arm of chromosomes 13 and 20, two chromosomal regions with gain/loss alterations associated with adenoma-to-carcinoma progression. Finally, the expression of the eight-genes signature and MSI-enriching genes was confirmed in oxaliplatin- and irinotecan-resistant CRC cell lines. These data reveal that the hypermethylation of specific genes may provide prognostic information that is able to identify a subgroup of mCRCs with poor prognosis.

## 1. Introduction

Colorectal carcinoma (CRC) is among the most frequent causes of cancer-related death in Western countries [1] and, despite significant improvements in treatment strategies, the prognosis of metastatic CRC (mCRC) remains poor [2]. First-line therapy includes either chemotherapeutics (i.e., fluoropyrimidines, oxaliplatin, irinotecan) or molecular-targeted agents and standard regimens are based on doublet- or triplet-chemotherapy regimens (i.e., FOLFOX, XELOX, FOLFIRI, and FOLFOXIRI) combined with antiangiogenic (i.e., bevacizumab) or anti-Epidermal Growth Factor Receptor (EGFR) (i.e., cetuximab or panitumumab) monoclonals. However, the main cause of treatment failure is drug resistance, and currently, a major clinical issue is tumor molecular profiling to improve our capacity to predict patients’ prognosis and design personalized treatments.

At present, among several proposed biomarkers, NRAS, KRAS, and BRAF mutational status and microsatellite instability (MSI) are the most reliable tools in clinical setting, allowing the selection of RAS/BRAF wild-type tumors that are more likely to respond to anti-EGFR agents [3,4] and MSI tumors that are more likely to respond to immune checkpoint inhibitors. No biomarkers are available to predict resistance/sensitivity to first-line chemotherapy and antiangiogenic agents.

For a long time, genetic aberrations and mutations in oncogenes and tumor suppressor genes have been considered the only molecular events driving tumor initiation and progression. Nowadays, epigenetic alterations gained consideration as additional crucial events in the multistep carcinogenetic process [5,6]. Indeed, the emerging leaning suggests a crosstalk between gene mutations and epigenetic alterations [5], and this interplay is responsible for the activation of signaling pathways regulating cancer hallmarks with an impact on clinical outcomes. Particularly, the majority of human cancers is characterized by mutations in enzymes (i.e., writers, readers, and erasers) involved in chromatin organization; hence, tumor cells are triggered by epigenetic alterations [7,8], and this results in the loss and gain of functions in genes correlated with tumorigenesis [9], drug resistance, and stem cell differentiation [10]. DNA methylation is the first epigenetic mechanism reported in humans [11,12,13], and the evaluation of DNA methylation of CpG island promoters represents the starting point of many cancer studies in this field. Moreover, since methylation remodeling is a rapid event compared to genetic mutations, it is likely that cancer cells preferentially use this mechanism to rapidly adapt to unfavorable conditions and trigger survival pathways, and this is particularly relevant in acquired and de novo resistance to anticancer agents [14]. Hence, a novel frontier for biomarker development is the identification of gene methylation patterns to predict clinical outcome, thus driving the selection of patients who may benefit from specific anticancer treatments. In such a context, this study examined the DNA methylation pattern of a cohort of primary-resistant mCRCs in comparison with drug-sensitive tumors treated with 1st-line FOLFOX or FOLFIRI backbone chemotherapy to identify epigenetic modifications able to predict patient’s prognosis.

## 2. Results

### 2.1. DNA Methylation Profile Is Remodeled in Primary-Resistant mCRCs

**Figure 1:**
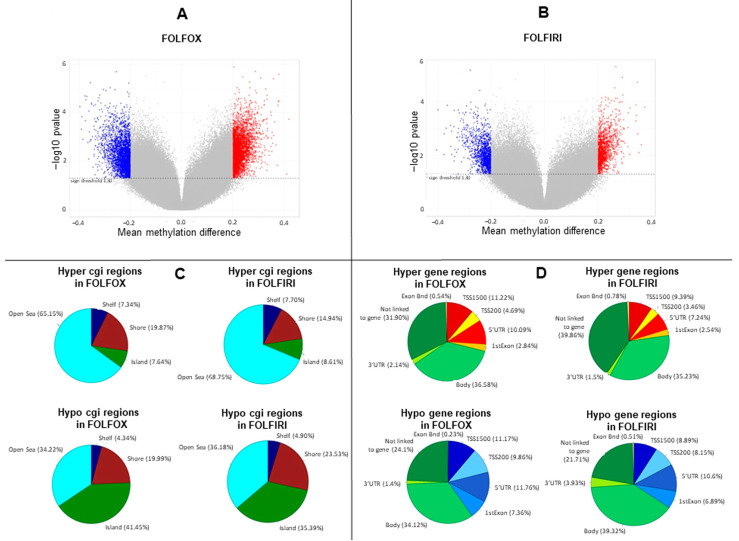
Methylation profile is remodeled in primary-resistant versus drug-sensitive metastatic colorectal carcinomas (mCRCs). (**A**,**B**). Volcano plots representing differentially methylated probes between primary-resistant and drug-sensitive mCRCs. Overall, statistically significant differentially methylated probes were 74,843 and 36,876 in, respectively, FOLFOX and FOLFIRI datasets (*p*-value < 0.05). In particular, statistically significant probes with an absolute difference of Beta value > 0.2 are highlighted as blue dots, corresponding to hypomethylated probes (3227 in FOLFOX and 1475 in FOLFIRI datasets), or as red dots, corresponding to hypermethylated probes (3899 in FOLFOX and 1393 in FOLFIRI datasets). (**C**,**D**). Differentially methylated probes distribution according to genomic regions.

Primary-resistant mCRCs were selected for this study as representative colorectal malignancies with poor prognosis and poor response to anticancer agents [15]. Thus, in order to identify epigenetic alterations with prognostic relevance, global DNA methylation was assessed on 24 mCRCs primary-resistant to 1st-line FOLFOX (16 patients) or FOLFIRI (eight patients) chemotherapy combined or not with molecular targeted agents. Twelve drug-sensitive mCRCs (four treated with FOLFOX and eight treated with FOLFIRI combined with molecular targeted agents) were used as controls to obtain the differential methylation profile between primary-resistant and drug-sensitive tumors (in-house cohort; Table S1). Differential methylation profiles were analyzed in a multistep process, as described in Figure S1. Indeed, 74,843 and 36,876 probes were significantly differentially methylated in, respectively, FOLFOX and FOLFIRI datasets (*p*-value < 0.05) (Figure 1A,B), and these were widely distributed between different genomic regions (Figure 1C,D).

### 2.2. Epigenetic Alterations Predict Prognosis in Human mCRCs

Since it is well established that promoter hypo/hypermethylation is the main mark resulting in gene expression modifications [16], only genes with methylation modifications in promoter regions (with a *p*-value < 0.05) were used in subsequent analyses. In particular, 19,454 probes, corresponding to 9760 genes, for patients treated with 1st-line FOLFOX and 10,892 probes, corresponding to 7218 genes, for patients treated with 1st-line FOLFIRI, resulted differentially methylated between drug-resistant and drug-sensitive tumors (Supplementary Materials Dataset S1).

We next questioned whether these DMGs were also functionally methylated (fMET), with a methylation profile consistent with the gene expression profile. Since we could not obtain gene expression data from in-house colorectal tumor samples due to the poor amount and quality of RNA purified from paraffin-embedded specimens, this issue was addressed using a cohort of 33 mCRCs obtained from The Cancer Genome Atlas COlon ADenocarcinoma (TCGA COAD) database, which provides gene expression, DNA methylation, DNA sequencing, and clinical data for each patient (Table S2). The result from the intersection of methylation data from TCGA and the in-house cohorts resulted in 7341 probes, corresponding to 4494 genes, for patients treated with 1st-line FOLFOX and 4961 probes, corresponding to 3774 genes, for patients treated with 1st-line FOLFIRI. We labeled these two lists FOLFOX differentially methylated genes (DMGs) and FOLFIRI DMGs, respectively (Supplementary Materials Dataset S2).

From the analysis of gene expression and methylation TCGA COAD data, we obtained 741 fMET genes defined as COAD fMET genes. Among these 741 TCGA COAD fMET genes, 542 were DMGs in the FOLFOX dataset and 248 were in the FOLFIRI dataset (Supplementary Materials Dataset S3), and 49 of them were common to both datasets. Applying more stringent filters (*p*-value < 0.01 and absolute difference of beta value > 0.2) on the FOLFOX_DMGs, we obtained 55 probes further restricted to eight hypermethylated fMET genes when selecting the COAD fMET genes with R2 > 0.5.

On the 248 genes of the FOLFIRI dataset, we applied a bit more relaxed filter (*p*-value < 0.05 and absolute difference of beta value > 0.1) obtaining 143 genes further restricted to 20 hypermethylated fMET genes (R2 > 0.5) (Supplementary Material Dataset S3). In order to produce two signatures, we performed an initial differential analysis on the COAD TCGA mCRCs dataset using, respectively, eight and 20 fMET genes and retaining only five and four genes differentially methylated with a greater stringency (see methods) (Table S3).

**Figure 2:**
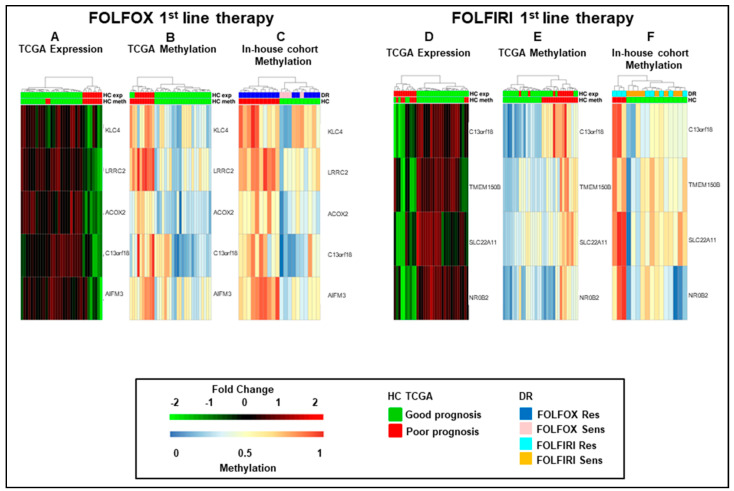
Hierarchical clustering of mCRCs according to the five and four-gene signatures. (**A**–**F**). Heatmaps of functionally methylated genes in The Cancer Genome Atlas COlon ADenocarcinoma (TCGA COAD) dataset (**A**,**B**,**D**,**E**) and in in-house first-line FOLFOX (**C**) or FOLFIRI (**F**) datasets. (**A**) and (**D**). Differential gene expression profiles in TCGA COAD. (**B**) and (**E**). Differential methylation profiles in TCGA COAD dataset. (**C**) and (**F**). Differential methylation profiles in in-house FOLFOX or FOLFIRI cohorts. HC, hierarchical clustering; DR, drug response.

Interestingly, hierarchical clustering on these sets of genes (using both expression and methylation data) allowed us to separate the TCGA COAD samples into two quite homogeneous clusters characterized by over or under expression (Figure 2A,D) and hypo or hyper methylation (Figure 2B,E) of, respectively, the above five and four fMET genes. A similar separation was obtained in our in-house FOLFOX and FOLFIRI cohorts upon hierarchical clustering of methylation data using the same gene sets (Figure 2C,F). In order to evaluate the prognostic relevance of these five and four fMET gene signatures, a log-rank test was performed on both the TCGA COAD and the in-house cohorts using the two previously obtained clusters. Noteworthy, with the exception of Relapse-Free Survival (RFS) using FOLFOX gene expression data, a significant (*p*-value < 0.05) separation for Event-Free Survival (EFS), Overall Survival (OS), and RFS curves was observed between hypermethylated/underexpressed tumors, which were characterized by worst prognosis, and hypomethylated/overexpressed tumors using the five-gene FOLFOX signature (Figure S2A–F). Consistently, OS and EFS curves were significant using the four-gene FOLFIRI signatures (Figure S3A–F). A log-rank test performed on in-house FOLFOX (Figure S4A,B) and FOLFIRI (Figure S4C,D) cohorts resulted in a significant (*p*-value < 0.05) separation of the two clusters considering RFS and a non-significant separation considering OS. Based on this evidence, the two cohorts were labeled as “good” and “poor” prognosis clusters.

**Figure 3:**
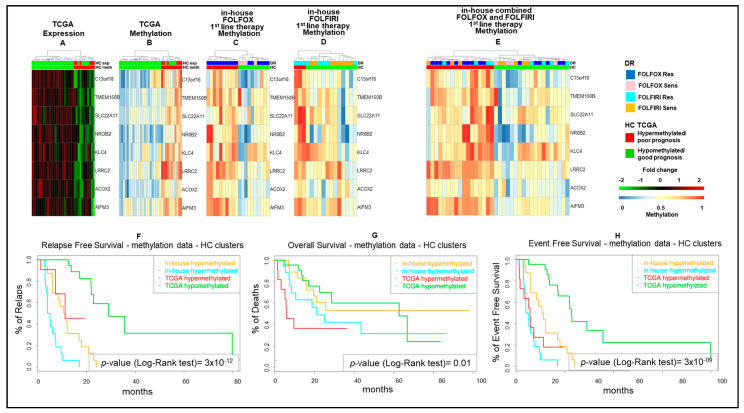
Hierarchical clustering and Kaplan–Meyer survival curves of mCRCs according to the eight-gene signature. (**A**–**E**). Heatmaps of functionally methylated (fMET) genes in TCGA COAD dataset (**A**,**B**) and in 1st-line FOLFOX (**C**), FOLFIRI (**D**) or combined FOLFOX/FOLFIRI (**E**) in-house datasets. (**A**,**B**). Differential gene expression (**A**) and methylation (**B**) profiles in TCGA COAD. (**C**–**E**). Methylation profiles in in-house cohorts. (**F**–**H**). Relapse-Free (**F**), Overall (**G**), and Event-Free (**H**) survival curves according to TCGA COAD or in-house clusters, as reported in (**A**,**B**,**E**). HC, hierarchical clustering; DR, drug response.

As a next step, we combined the five and four fMET gene signatures from FOLFOX and FOLFIRI datasets obtaining a new signature of eight fMET genes, being one of them common to both datasets (Figure 3). Hierarchical clustering using such signature separated TCGA COAD patients into two well-defined cohorts (22 hypo and 11 hypermethylated tumors) (Figure 3A,B and Table S2). A similar clustering was obtained in our in-house FOLFOX dataset (Figure 3C) and partially in the FOLFIRI dataset (Figure 3D). Furthermore, upon combination of in-house FOLFOX and FOLFIRI datasets, the eight-gene signature obtained a separation between 19 hypo and 17 hypermethylated tumors (Figure 3E). It is noteworthy that the hypomethylated cluster contains a tight sub-cluster of six drug-sensitive samples characterized by strongly hypomethylated genes. The log-rank test confirmed that the cohort with hypermethylation of the eight-gene signature is characterized by significantly shorter survival (RFS, OS, and EFS; *p*-value < 0.05) compared to the cohort with hypomethylation of these genes in both TCGA COAD and in-house cohorts (Figure 3F–H). Altogether, these data suggest that this eight-fMET gene signature discriminates between mCRC patients with good (hypomethylated tumors) and poor (hypermethylated tumors) prognosis.

### 2.3. Differential Epigenetic Alterations Obtained According to the Eight-Gene Signature Are Similar in in-House, TCGA, and GSE48684 Datasets

To strengthen our results, we retrieved another dataset, the GSE48684 from NCBI GENE expression Omnibus repository (GEO), which provided the methylation profile of 24 mCRCs. Upon clustering the GSE48684 samples according to the eight-gene signature, we observed two homogeneous methylation clusters (Figure S5A), as previously reported for the COAD TCGA and the in-house cohorts. Thus, we performed differential methylation analysis on the GSE48684, the TCGA, and the whole in-house datasets, obtaining a very significant overlap (*p*-value < 0.01) between the lists of DMGs in the three cohorts (Supplementary Materials Dataset S4). In parallel, Gene Set Analysis (GSA) of the same three datasets exhibited a significant overlap between the enrichments of the collections retrieved from the mSigDB repository (Supplementary Materials Dataset S5–S7 and Figure S5B). Altogether, these data confirm the significance of the epigenetic reprogramming observed in the in-house FOLFOX/FOLFIRI primary-resistant cohort.

### 2.4. The Poor Prognosis Hypermethylated Cluster Is Characterized by an MSI-Like Phenotype and Is Enriched of CIMP-High Tumors

The poor prognosis hypermethylated and the good prognosis hypomethylated clusters were further characterized with respect to their clinical and biological profiles using gene expression and DNA sequencing and gene copy number data from the TCGA COAD database. No major differences were observed between the poor and good prognosis clusters with respect to T and N categories and sites of primary tumor (right versus left colon) (Table S2). Similarly, no major differences were observed with respect to the tumor mutational load, with the exception of two hypermutated cases in the poor prognosis cluster (Figure S6, insert). Interestingly, specific gene mutations were differently distributed between the two subgroups, being mutations in SRGAP2B, AC007682.1, AC104820.2, and AF121898.3 genes enriched in the good prognosis cluster and mutations in the GRP98, NRXN2, HDN1, and TTC40 genes in the poor prognosis cluster (Figure S6). Consistently, several gene aberrations were significantly more abundant in the good prognosis cluster (Figure S7).

Considering that a slight difference was observed in the hierarchical clustering of TCGA mCRCs according to the eight-gene signature using gene expression or methylation data (Figure 3A,B), we produced two lists of DEGs, one for each clustering, and tested these lists of genes for the significance of the overlap. As expected, the two DEGs lists are significantly overlapped (Supplementary Materials Dataset S8); thus, for further analyses, we decided to use methylation clusters. A differential gene expression comparison between hypermethylated and hypomethylated tumors yielded 444 Differentially Expressed Genes (DEGs) (false discovery rate, FDR, adjusted *p*-value < 0.05 and abs(logFC) > 0.58). Among these, 307 genes were downregulated and 137 were hypermethylated in the poor prognosis cluster and conversely upregulated/hypomethylated in the good prognosis subgroup (Figure S8). GSA was performed on the gene set collection of the mSigDB repository, obtaining significant enrichments for signaling pathways and positional collections.

**Figure 4:**
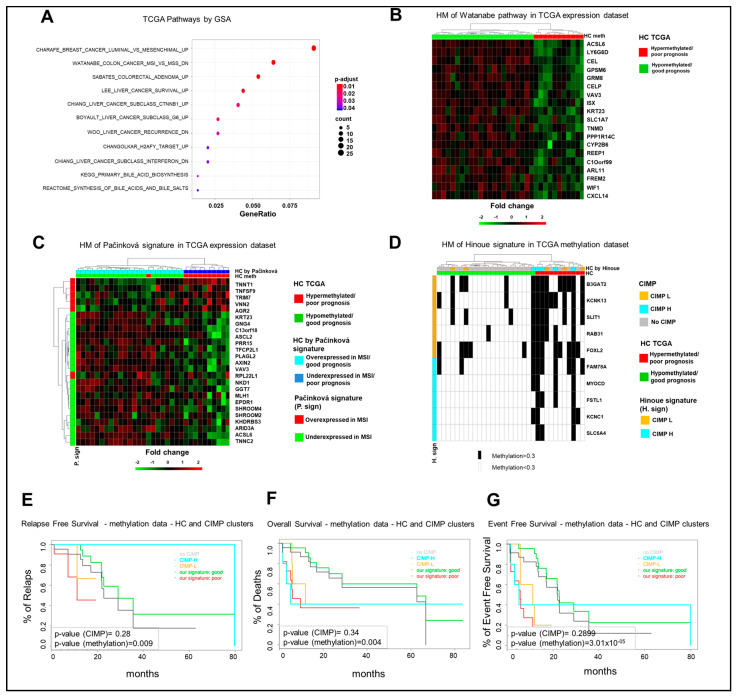
The characterization of the poor prognosis hypermethylated cluster highlights a microsatellite instability (MSI)-like/CpG-island methylator-high (CIMP-H) phenotype. (**A**). Significant enrichments for signaling pathways upon Gene Set Analysis (GSA). (**B**). Heatmap (HM) of differentially expressed genes enriching the Watanabe gene set in 33 mCRCs from the TCGA COAD dataset. (**C**). Heatmap of differentially expressed genes from the MSI-like gene expression Pačínková signature in 33 mCRCs from the TCGA COAD database. (**D**). Heatmap of CIMP status in 33 mCRCs from the TCGA database according to the eight-gene signature. CIMP status is labeled in black. (**E**–**G**). Relapse-Free, Overall, and Event-Free survival curves of TCGA COAD patients according to the eight-gene signature or CIMP status. HC, hierarchical clustering; DR, drug response.

Among different signaling pathways (Figure 4A), GSA identified the Watanabe gene set, which includes genes discriminating between MSI and MSS (microsatellite instability/stability) colorectal cancers [17]. The statistical analysis identified 19 genes in our list of DEGs, which enrich the Watanabe gene dataset and whose expression profile is consistent with a separation of the TCGA cohort in good and poor prognosis clusters (Figure 4B). Noteworthy, the Watanabe pathway result was significantly enriched (*p*-value < 0.01) in the eight-gene hypermethylated cluster of the GSE48684 and in-house datasets (Figure S9A,B). Since these observations suggest that the eight-gene hypermethylated signature identifies a subgroup of mCRCs with an MSI-like phenotype, an independent MSI-like gene expression signature was evaluated for the capacity to reproduce the separation of the TCGA COAD cohort in the same good and poor prognosis clusters, according to the eight-gene signature. Noteworthy, the MSI-like gene expression signature of Pačínková et al. [18] mirrored the separation of the 33 mCRCs TCGA cohort in the same clusters as obtained by our eight-gene signature (Figure 4C). Consistently, 11 genes from the Pačínková signature were characterized by an expression profile consistent with the expression profile of the MSI-like poor prognosis TCGA cluster.

Interestingly, 15–20% of human CRCs are characterized by the CpG-island methylator phenotype (CIMP), subdivided in CIMP-high (CIMP-H) and CIMP-low (CIMP-L), and this correlates with the MSI phenotype [19]. Thus, the relationship between our eight-gene hypermethylated signature and CIMP status was evaluated in the TCGA COAD dataset and in our in-house cohort according to Hinoue et al. [20]. Of note, the poor prognosis TCGA hypermethylated cohort was enriched of CIMP-H cases, being four out of five CIMP-H samples classified as poor prognosis patients, whereas the hypomethylated good prognosis cohort was enriched on no-CIMP tumors. CIMP-L cases were distributed between the two subgroups (Figure 4D). This difference between the groups was statistically significant by a two-sided Fisher exact test (*p*-value < 1 × 10^−2^). In our in-house cohort, while all the CIMPs belong to FOLFOX/FOLFIRI-resistant samples, they divide evenly between the eight-gene signature clusters (Figure S10A). Finally, six of nine CIMP tumors belong to the hypermethylated cluster in the 24 GSE48684 mCRC samples (Figure S10B). Thus, the prognostic relevance of our eight-gene signature was compared to CIMP status and, noteworthy, RFS, OS, and EFS curves showed a better capacity of our eight-gene hypermethylated signature to predict poor prognosis in the TCGA COAD dataset (Figure 4E–G). Altogether, these observations strongly support the conclusion that the eight-gene hypermethylated signature correlates with an MSI-like phenotype and is characterized by a better capacity to predict prognosis compared to CIMP status.

### 2.5. Hypermethylated Genes Are Enriched on Arms q of Chromosomes 13 and 20

**Figure 5:**
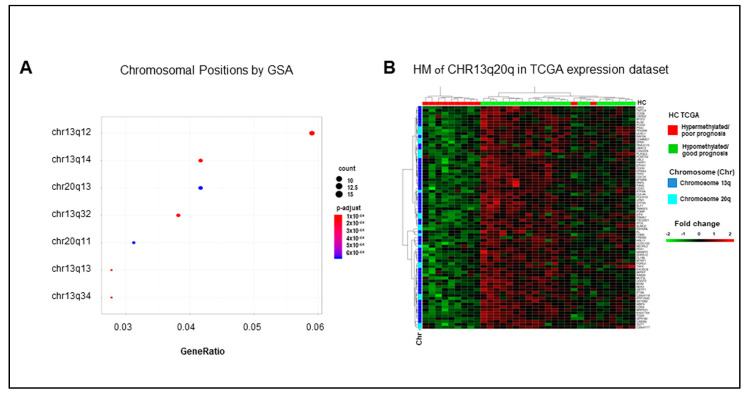
The poor prognosis hypermethylated cluster is enriched of genes located on arm q of chromosomes 13 and 20. (**A**). Significant enrichments for the genomic positional collections upon GSA analysis. (**B**). Heatmap (HM) of differentially expressed genes enriching chromosome 13 arm q and chromosome 20 arm q gene sets in 33 mCRCs from the TCGA COAD dataset.

Among positional collections, GSA identified enrichments of chromosome 13 arm q and chromosome 20 arm q gene sets (Figure 5A). Interestingly, 56/307 downregulated genes in our list of DEGs are located on chromosome 13 arm q, and 17 of them are hypermethylated. Consistently, 21/307 downregulated genes are located on chromosome 20 arm q, seven of them hypermethylated. It is noteworthy that the expression profile of each of these gene sets (Figure S11A,B) and of their combination (Figure 5B) mirrored the separation of the TCGA COAD cohort in the good and poor prognosis clusters, which were obtained according to the eight-genes signature. These data suggest an enrichment of methylation events in genes located in specific chromosomal regions in mCRCs with poor prognosis. In such a context, a slightly relaxed enrichment analysis for signaling pathways identified the Ding lung cancer expression by copy number (adjusted *p*-value 0.08) and the Nikolsky breast cancer 20q12-13 amplicon (adjusted *p*-value 0.09) gene sets. These authors reported respectively a correlation between the copy number variation and the expression of 26 genes in lung cancers [21] and the identification of 149 genes in amplicon 20q12-13 in breast tumors [22]. As expected, all the genes enriching the Nikolsky gene set overlap with our chromosome 20 arm q genes, as well as Ding genes overlap with our chromosome 13 arm q genes. In both cases, the expression profile of these genes reproduced the separation of the TCGA dataset in good and poor prognosis clusters (Figure S11C,D). Altogether, these data highlight the relevance of expression/methylation modifications of genes located on chromosomes 13 and 20 in human colorectal cancer.

### 2.6. Epigenetic Modifications Are Reproduced in Drug-Resistant Cell Models

**Figure 6:**
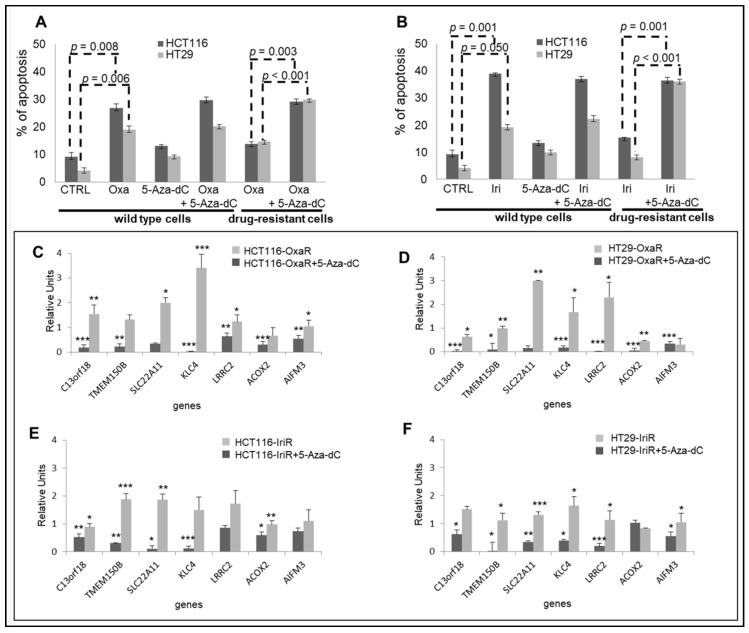
Validation of the eight-gene signature in drug-resistant CRC cell lines. (**A**,**B**). Apoptotic cell death in HCT116 and HT29 drug-sensitive and drug-resistant CRC cell lines exposed to 10 uM 5-Azacytidine (5-Aza-dC) for 48 h and/or 3 uM oxaliplatin (Oxa) (**A**) or 2uM irinotecan (Iri) (**B**) for 24 h. (**C**–**F**). Real-time differential expression analysis of eight genes belonging to the prognostic signature between drug-resistant and drug-sensitive CRC cell lines before and after exposure to 10 uM 5-Aza-dC for 48 h. C. HCT116-OxaR: D. HT29-OxaR; E. HCT115-IriR; F, HT29-IriR. Significantly modulated genes are indicated by asterisks: * = *p* < 0.05; ** = *p* < 0.01; *** = *p* < 0.001. Apoptosis and PCR analyses were performed in triplicate.

To validate epigenetic data obtained from FOLFOX and FOLFIRI primary-resistant mCRCs, we generated in vitro drug-resistant cellular models chronically adapted to oxaliplatin (Oxa; HCT116-OxaR and HT29-OxaR cells) or irinotecan (Iri; HCT116-IriR and HT29-IriR cells). In preliminary experiments, apoptotic cell death was evaluated in drug-sensitive and drug-resistant cell lines in response to Oxa or Iri in combination or not with the demethylating agent 5-Azacytidine (5-Aza-dC). These experiments confirmed that cell lines chronically exposed to chemotherapeutics are indeed poorly sensitive to Oxa and Iri and that drug resistance is reverted upon treatment with 5-Aza-dC (Figure 6A,B). Since these data support the hypothesis that methylation modifications are responsible for resistance to Oxa and Iri in these CRC cell lines, in subsequent experiments, drug-resistant cell lines were used to validate the expression profiles of the eight-fMET gene signature. Real-Time RT-PCR analysis of the eight genes confirmed a significant downregulation of six genes in HCT116-OxaR, HT29-OxaR, and HT29-IriR cell lines (Figure 6C,D,F) and five genes in HCT116-IriR cell lines (Figure 6E) compared to the respective drug-sensitive cell lines. NR0B2 was undetectable in both cell lines. It is noteworthy that the pretreatment of drug-resistant cell lines by 5-Aza-dC resulted in a significant upregulation of the majority of the downregulated genes: six genes in HT29-OxaR cell line (Figure 6D), five genes in, respectively, HCT116-OxaR and HT29-IriR (Figure 6C,F), and four genes in HCT116-IriR (Figure 6E). These data suggest that the downregulation of genes belonging to the eight-gene signature correlates with the onset of drug resistance in CRC cell lines and that this downregulation is likely mediated by methylation events. In parallel analyses, starting from GSA results suggesting an enrichment of an MSI-like phenotype in hypermethylated poor-prognosis tumors, seven representative genes enriching the Watanabe pathway and five representative genes belonging to the mismatch repair system [18] were evaluated in drug-resistant cell lines (Figure S12). Indeed, PCR data confirmed the downregulation of the majority of these genes in drug-resistant cell lines with HT29-OxaR and HT29-IriR characterized by a more evident MSI-like phenotype (Figure S12).

## 3. Discussion

Molecular profiling is a major objective in mCRC in order to define prognostic homogeneous subgroups of patients and deliver personalized therapies [23]. In such a context, genetic mutations and gene expression profiles have been proposed as potential predictive/prognostic biomarkers and some of them have entered in daily clinical practice [24,25]. By contrast, while preclinical evidence is currently available on the role of epigenetic modifications in tumor progression [26], their significance as prognostic tools is so far mostly unexplored and/or results are conflicting [27,28]. Indeed, the majority of human CRCs are characterized by global hypomethylation and promoter-specific DNA methylation [29], whereas 15–20% of them exhibit the CIMP status, with extensive and co-ordinate patterns of hypermethylation events in numerous CpG islands surrounding the promoter regions of several genes whose transcriptional silencing contributes to the onset and progression of CRC [30,31]. The prognostic significance of CIMP is controversial, with several studies suggesting that CIMP status is an independent prognostic factor of poor outcome [32,33]. However, this conclusion is still debated, which is likely due to different definitions of CIMP among studies with respect to methylation loci and laboratory methods [32]. Recently, in contrast with the conclusion that CIMP status predict poor prognosis, a prognostic score based on the low methylation level of seven CpG sites was strongly associated with poor CRC survival [32]. Thus, in order to better define the prognostic relevance of promoter hypermethylation events in human CRC, this study was designed to characterize epigenetic signatures helpful to identify mCRCs molecular subgroups with defined clinical behavior. In such a context, primary-resistant colorectal carcinomas were selected as cases representative of poor outcome [33] based on the evidence that methylation modifications are key events used by cancer cells to rapidly adapt to unfavorable environments and acquire drug resistance [34,35]. Our data suggest that the methylation profile of eight functionally methylated genes is predictive of clinical outcome being able to clusterize two independent mCRC cohorts (i.e., the TCGA COAD and the in-house datasets) in two well-defined clusters with hypermethylated tumors characterized by worse prognosis and an MSI-like phenotype compared to hypomethylated tumors. As expected, the hypermethylated poor prognosis cluster is enriched with CIMP-H cases in the TCGA COAD cohort, but our eight-gene signature showed a better capacity to identify hypermethylated malignancies with poor prognosis compared to CIMP status. Clinically relevant is the observation that the poor prognosis cohort with hypermethylation of the eight-gene signature is characterized by an MSI-like phenotype. Consistently with this conclusion, the eight-gene signature was able to identify a cluster of hypermethylated mCRCs enriched of MSI-like and CIMP-H cases in a third independent dataset (GEO GSE48684). 

The CIMP-H status is frequently associated with the methylation of MLH1 promoter region and consequent gene silencing [20], resulting in the acquisition of MSI and strong immune activation [36]. In such a context, the relevance of the MSI phenotype is a controversial issue in CRCs. Indeed, TNM stage II colorectal tumors with deficient mismatch repair system/MSI-high phenotype are characterized by good prognosis, but they do not benefit from 5-fluorouracile adjuvant chemotherapy [37]. By contrast, there are several controversies about whether the MSI-high phenotype is a good prognostic factor in mCRC patients. Some studies proved that MSI-high is a beneficial factor associated with a better outcome [38,39], whereas several others came to the opposite conclusion, indicating MSI-high as an adverse factor [40,41]. On the other hand, unlike MSS CRCs, MSI-high CRCs showed a much better response to immune checkpoint inhibitors [42,43]. More recently, several MSI-like gene expression signatures were also proposed with likewise controversial significance [18,44,45]. Our data suggest that the eight-gene hypermethylated cohort of mCRCs is characterized by an MSI-like phenotype and that the methylation profile of the eight-gene signature may represent an alternative strategy to better define a subgroup of mCRCs with a CIMP-H status and an MSI-like phenotype, which is characterized by a poor clinical outcome. In such a context, a limitation of our study is the limited number of cases in our series, even though the biological and the clinical significance of the eight-gene signature was proven in, respectively, three and two independent mCRC datasets. However, its prognostic relevance needs to be further validated in a larger series to establish its wider use in a clinical setting, and further studies are also needed to establish whether this gene signature may improve our capacity to select mCRCs amenable to immunotherapy. In a biological perspective, it is noteworthy that two genes in our list, C13orf18 and LRRC2, are putative oncosuppressor genes. Indeed, C13orf18, a gene with a hypermethylation status common to both FOLFOX and FOLFIRI first-line datasets, is frequently hypermethylated and silenced in cervical cancer, and its re-expression results in the growth inhibition of cervical tumor cells [46]. In addition, C13orf18 is significantly downregulated in our drug-resistant cell lines, and its expression is reverted by the demethylating treatment. LRRC2 gene expression is impaired in renal carcinoma cell lines, this also suggesting a putative oncosuppressive gene function [47]. Thus, the functional hypermethylation of both these genes in a cohort of mCRCs with poor outcome supports the hypothesis that these genes may play a role in colorectal carcinogenesis.

Finally, GSA enrichment analysis suggests that methylation events are enriched in genes located on arm q of chromosomes 13 and 20 in mCRCs with poor prognosis, supporting the hypothesis that epigenetic remodeling may not occur in a random manner during colorectal carcinogenesis, but rather, it may be a coordinated process with the hypo/hypermethylation of selective genomic regions. This hypothesis is consistent with the evidence that the accumulation of gains/losses in 13q and 20q regions is strongly associated with adenoma-to-carcinoma progression [48] and that mutations in the same chromosomal regions are relevant in other human malignancies. Indeed, the GSA identified the Ding lung cancer expression by copy number and the Nikolsky breast cancer 20q12-13 amplicon gene sets. Ding et al. reported a correlation between the copy number variation and the expression of 26 genes in lung cancers [21], whereas Nikolsky et al. reported the identification of 149 genes in amplicon 20q12-13 in breast tumors [22]. It is intriguing that genes enriching Ding and Nikolsky gene sets reproduced the clustering of TCGA mCRCs in the same cohorts as obtained by the eight-gene signature. Consistently, the vast majority of genes enriching the Nikolsky and the Ding gene sets overlap with our chromosome 20 arm q or 13 arm q genes.

In conclusion, this study provides the proof of concept that epigenetic profiling may represent a strategy to predict patients’ prognosis in mCRC and that a novel eight-gene methylation signature may better define a poor prognosis subgroup of mCRCs with CIMP-H status and an MSI-like phenotype.

## 4. Materials and Methods

### 4.1. Patients and Samples Collection

Twenty-four primary-resistant mCRCs treated with 1st-line FOLFOX (16 patients) or FOLFIRI (8 patients) chemotherapy in combination or not with bevacizumab or anti-EGFR agents and 12 drug-sensitive mCRCs (4 treated with FOLFOX and 8 treated with FOLFIRI combined with molecular targeted agents) were selected for this study. Patients’ characteristics are described in Table S1. Tumors were selected based on the evidence of tumor progression (primary-resistant) or partial/complete response (drug-sensitive) at the first radiological assessment after 2–3 months of first-line therapy. Patients were enrolled at the Medical Oncology Units of the IRCCS-CROB (Rionero in Vulture, Italy) and the Fondazione Policlinico Universitario “A. Gemelli” (Rome, Italy) and were called the “in-house” cohort. All experiments were performed in accordance with protocols approved by Ethics Committee of IRCCS CROB (reference number 20120010288). Express written informed consent to use biological specimens for investigational procedures was obtained from all patients.

### 4.2. Cell Lines and In Vitro Drug-Resistant Models

Human HT29 and HCT116 CRC oxaliplatin and irinotecan-resistant cell lines were obtained in our laboratory as described in Supplementary Information. Experiments were carried out at 70% cell confluence and confirmed at least in three independent replicates. Cell cultures were routinely screened for mycoplasma contamination.

### 4.3. Array-Based DNA Methylation Profiling

DNA was isolated from formalin-fixed, paraffin-embedded (FFPE) primary CRCs (Supplementary Information). Five hundred ng of total gDNA were treated with sodium bisulfite using the Zymo EZ DNA Methylation Kit (Zymo Research, Irvine, CA, USA) according to the Infinium HD Methylation Assay protocol. The bisulfite converted gDNA was hybridized on the Infinium Human Methylation 850 BeadChip array (Illumina Inc., San Diego, CA, USA), following the manufacturer’s instructions. After washing and staining procedures, chips were scanned by the Illumina HiScanSQ system.

### 4.4. Bioinformatics Analysis

To identify a prognostic signature of fMET genes, a multistep analysis was performed as described in Figure S1. In the first step, global DNA methylation profiles were obtained from primary-resistant and drug-sensitive in-house tumor specimens [49], as described in Supplementary Information.

In a subsequent step, since gene expression data from in-house tumors were not available, fMET genes were defined based on gene expression and methylation data from the TCGA COAD data collection. To this purpose, the gene expression, methylation, DNA sequencing, gene copy number, and clinical data of 33 patients with mCRC from the TCGA COAD database were downloaded using the TCGA biolink package (Table S2).

Moreover, the methylation dataset GSE48684, containing 24 stage IV mCRCs, was used to validate our methylation and GSA data.

All differential analyses were performed applying linear modeling (limma) to the log2 ratio of the intensities of methylated versus unmethylated probe (from now on M-value) datasets. We also calculated the beta value as the ratio of the methylated probe intensity and the overall intensity.

The DMGs from the TCGA, GSE48684, and in-house datasets were all filtered at a *p*-value < 0.05 and absolute difference of beta value > 0.1, if not reported differently.

Annotation of the methylation datasets is according to illumina R packages. In particular, being the TCGA and the GSE48684 dataset based on Illumina 450k technology, we reduced the considered probes of our in-house dataset to such annotations to make the comparisons meaningful.

The overlap between two DMGs or enriched gene sets was simply evaluated by a hypergeometric test, while the overlap among three or more of them was based on a more complex framework implemented in the R SuperExactTest package [50].

We defined as fMET genes those showing a significant (FALSE DISCOVERY RATE, FDR, adjusted *p*-value < 0.05), and inverse correlation (R Squared > 0.1) between the promoter’s methylation and the gene expression profile in the TCGA dataset. Among these, we selected only the DMGs in the FOLFOX or in the FOLFIRI dataset.

For the FOLFOX in-house dataset, given the 8 fMET genes, we performed a hierarchical clustering on the TCGA mCRCs datasets (Mvalues) obtaining two clusters, one grouping the samples with the hypermethylated genes signature and the other with the hypomethylated gene signature. Finally, we checked if the 8 genes were differentially methylated (*p*-value < 0.05 and either BetaFC > 0.2 or logFC > 1), obtaining the final 5-gene signature. The same procedure was performed for the 20 FOLFIRI fMET genes, obtaining the final 4-gene signature. Hierarchical clustering was performed using Ward’s linkage and Euclidean distance.

To evaluate differences in prognosis, Kaplan–Meier estimator and log-rank tests were applied to both TCGA COAD and in-house overall, relapse, and event-free data. To identify biological and clinical differences between the identified clusters, a hypergeometric test for the enrichment analysis (GSA) was performed on all gene set collections of the mSigDB repository using CluterProfiler R package [51].

### 4.5. RNA Extraction and Real-Time RT-PCR Analysis

Total RNA was extracted using the TRIzol Reagent (Invitrogen, Whitby, CANADA) from parental and drug-resistant strains of HCT116 and HT29 CRC cells lines. In specific experiments, RNA was obtained from cell lines exposed to 5-Aza-dC (Sigma-Aldrich, Burlington, MA, USA), an inhibitor of DNA methyltransferase 1 (DNMT1), at a final concentration of 10 μM for 72 h. Real-Time PCR analysis was described in Supplementary Information. Primers are reported in Table S4.

### 4.6. Apoptosis Assay

Parental and drug-resistant cell lines were seeded on day 0 in 6-well plates in triplicate and incubated on day 1 in normal medium or exposed to 10 μM 5-Aza-dC for 72 h. After 48 h, cells were further treated with 3 μM Oxa or 2 μM Iri for 24 h. Apoptosis was evaluated by cytofluorimetric analysis (Supplementary Information).

### 4.7. Data Availability

DNA methylation data generated in this study have been deposited at the NCBI GENE expression Omnibus repository (GEO) and are accessible through the accession number GSE148766 (www.ncbi.nlm.nih.gov/geo/).

## 5. Conclusions

In spite of significant improvements in the treatment of mCRC, the prognosis remains still poor. Until today drug resistance is the main cause of treatment failure and the main issue is tumor molecular profiling to improve our capacity to predict patients’ prognosis. The data showed in this paper represent the proof of concept that the hypermethylation of specific sets of genes may provide prognostic information being able to identify a subgroup of mCRCs with poor prognosis.

## Data Availability

The data presented in this study are available in the article or supplementary files. DNA methylation data generated in this study have been deposited as stated above (Section 4.7).

## Supplementary Materials

The following are available online at https://www.mdpi.com/2072-6694/13/1/158/s1, Figure S1: Study flow chart, Figure S2: Kaplan–Meyer survival curves of mCRCs clusters according to the five-gene 1st-line FOLFOX signature in the TCGA COAD dataset, Figure S3: Kaplan–Meyer survival curves of mCRCs clusters according to the four-gene 1st-line FOLFIRI signature in the TCGA COAD dataset, Figure S4: Kaplan–Meyer survival curves of mCRCs clusters according to the five- and four-gene signatures in 1st-line FOLFOX and FOLFIRI in-house datasets, Figure S5: Validation panels, Figure S6: DNA mutational characterization of the poor and good prognosis cluster, Figure S7: DNA functionally gene aberrations in poor and good prognosis cluster, Figure S8: Heatmap (HM) of 444 differentially expressed genes (DEGs) between the TCGA hypermethylated/poor prognosis and hypomethylated/good prognostic clusters, Figure S9: Watanabe signature in GSE48684 and in-house datasets, Figure S10: The in-house tumors CIMP status, Figure S11: Hierarchical clustering according to enrichment analysis gene sets, Figure S12: Validation of Watanabe and mismatch repair genes in drug-resistant CRC cell lines, Table S1: Baseline patients’ characteristics of the in-house cohort, Table S2: Baseline patients’ characteristics of the TCGA cohort, Table S3: Functionally methylated genes obtained from the intersection of in-house and TCGA datasets, Table S4: Oligonucleotides utilized in Real-Time RT-PCR analysis, Supplementary Dataset S1: Differentially methylated promoter probes between primary-resistant and drug-sensitive mCRCs, Supplementary Dataset S2: Differentially methylated genes (DMGs) between drug-resistant and drug-sensitive tumors after TCGA intersection, Supplementary Dataset S3: COAD fMET genes, Supplementary Dataset S4: Overlap between the DMGs obtained in the three datasets, Supplementary Dataset S5: Overlap between the mSigDB gene sets enriched in in-house and GSE48684, Supplementary Dataset S6: Overlap between the mSigDB gene sets enriched in in-house and TCGA, Supplementary Dataset S7: Overlap between the mSigDB gene sets enriched in TCGA and GSE48684, Supplementary Dataset S8: Overlap between the mSigDB gene sets enriched in TCGA expression samples using methylation or expression based clustering.
