# Supplementary material for: Novel Epigenetic Eight-Gene Signature Predictive of Poor Prognosis and MSI-Like Phenotype in Human Metastatic Colorectal Carcinomas
Source: Cancers (Basel). 2021 Jan 5;13(1):158. doi: 10.3390/cancers13010158 (PMC7796477; doi:10.3390/cancers13010158)
Supplement: Supplementary file 1 [file cancers-13-00158-s001.zip › Supplementary information/Supplementary Information.pdf]

Supplementary Experimental Procedures: Novel Epigenetic Eight-Gene Signature Predictive of Poor Prognosis and MSI-Like Phenotype in Human Metastatic Colorectal Carcinomas

Valentina Condelli, Giovanni Calice, Alessandra Cassano, Michele Basso, Maria Grazia Rodriquenz, Angela Zupa, Francesca Maddalena, Fabiana Crispo, Michele Pietrafesa, Michele Aieta, Alessandro Sgambato, Giampaolo Tortora, Pietro Zoppoli and Matteo Landriscina

Cell culture.

Human HT29 and HCT116 CRC cell lines were obtained from the American Type Culture Collection (ATCC). Cell lines were routinely monitored in our laboratory by microscopic morphology, while cell line authentication was verified before starting this study by STR profiling, according to ATCC product description. Resistant strains were generated as previously descrived (1) from parental cell lines upon progressive drug dose escalation from 0.1 to 3 μM Oxa (Roche, Penzberg, Germania) and from 0.1 to 2 μM Iri (Roche, Penzberg, Germania) over a 40-days period. Cells lines were cultured in McCoy's medium supplemented with 10% (v/v) FBS, 0.75 mM glutamine and 10 U/mL penicillin and streptomycin. (GIBCO).

Genomic DNA Extraction from tumor specimens.

Representative tumor areas from formalin-fixed, paraffin-embedded (FFPE) CRC specimens were identified based on hematoxylin and eosin stain and cut into 6 10-μm-thick sections. Deparaffinized dried sections were used for genomic DNA (gDNA) extraction by using the QIAamp DNA Mini kit (Qiagen, Milano MI, Italia). DNA concentration and purity were assessed by NanoDrop technology (Thermo Scientific, Waltham, MA USA) using, respectively, the absorbance at 260 nm and the 260/280 nm absorbance ratio.

Microarray data analysis.

Illumina Infinium Human Methylation EPIC raw data were imported into R/Bioconductor environment (2) for quality control, normalization, visualization, and processing, according to Todoerti et al. (3). For graphic representation, the methylation level was expressed mainly in terms of β-value, which describes the ratio between methylated and total (methylated and unmethylated) signals (2), according to the following formula: β-value = M/(M+U+offset). For the differential analysis, the M-value was assessed, as log ratio between methylated and unmethylated signals (2), according to the following formula: M-value = log2(M/U). For the annotation of methylation probes we used the annotations for Illumina's EPIC methylation arrays (IlluminaHumanMethylationEPICanno.ilm10b2.hg19) package and we focused on genomic context (TSS1500, TSS200, 5'UTR, 1stExon, GeneBody, 3'UTR, ExonBnd and Not linked to gene) and CGI regions (Shelf, Shore, Island and Open Sea). The differential analysis between primary-resistant and drug-sensitive tumors provided a lists of Differentially Methylated Positions. Probes and corresponding genes were interpreted as differentially methylated if the *p*-value was < 0.05. Volcano plots and pie charts were obtained by the ggplot2 R package. For subsequent analyses, only methylation events occurring in promoter regions were considered, and a probe was interpreted as promoter if annotated as TSS1500, TSS200, 5'UTR or 1stExon in UCSC_RefGene_Group (Gene region feature category describing the CpG position, from UCSC) by Illumina Infinium MethylationEPIC Manifest.

Cytofluorimetric analysis.

Apoptosis was evaluated by cytofluorimetric analysis of Annexin-V and 7-amino-actinomycin-D (7-AAD)-positive cells using the fluorescent isothiocyanate (FITC)-Annexin-V/7-AAD kit (Beckman Coulter, Indianapolis, IN USA). Stained cells were analyzed using the FACSCaliburTM (Becton Dickinson, Franklin Lakes, NJ USA). Positive staining for Annexin-V and 7-AAD were interpreted as signs of early and late phases of apoptosis respectively. Apoptosis analysis was performed in triplicate.

Real Time RT-PCR analysis

The RNA concentration and purity were assessed by NanoDrop technology (Thermo Scientific, Waltham, MA USA) using, respectively, the absorbance at 260 nm and the 260/280 nm absorbance ratio. For first strand synthesis of cDNA, 1 μg of RNA was used in a 20 μL reaction mixture utilizing a Transcriptor First Strand cDNA Synthesis Kit (Roche, Penzberg, Germania). For Real Time PCR analysis, 0.5 ng of cDNA sample was amplified using the LightCycler 480 SYBR Green I Master (Roche, Penzberg, Germania) in a Light Cycler 480 (Roche, Penzberg, Germania). Reaction conditions were as follows: pre-incubation at 95 °C for 5 min, followed by 45 cycles of 10 s at 95 °C, 10 s at 60 °C, 10 s at 72 °C. ßActin was chosen as an internal control. Analyses were conducted in triplicate.

Supplementary References

1. Costantino E.; Maddalena F.; Calise S.; Piscazzi A.; Tirino V.; Fersini A.; et al. TRAP1, a novel mitochondrial chaperone responsible for multi-drug resistance and protection from apoptotis in human colorectal carcinoma cells. *Cancer Lett* **2009**, *279*, 39–46.
2. Aryee MJ.; Jaffe AE.; Corrada-Bravo H.; Ladd-Acosta C.; Feinberg AP.; Hansen KD.; et al. Minfi: a flexible and comprehensive Bioconductor package for the analysis of Infinium DNA methylation microarrays. *Bioinformatics* **2014**, *30*, 1363–1369.
3. Todoerti, K.; Calice, G.; Trino, S.; Simeon, V.; Lionetti, M.; Manzoni, M.; Fabris, S.; Barbieri, M.; Pompa, A.; Baldini, L.; et al. Global methylation patterns in primary plasma cell leukemia. *Leuk. Res.* **2018**, *73*, 95–102, doi:10.1016/j.leukres.2018.09.007.

Supplementary Figures and Supplementary Figure Legends

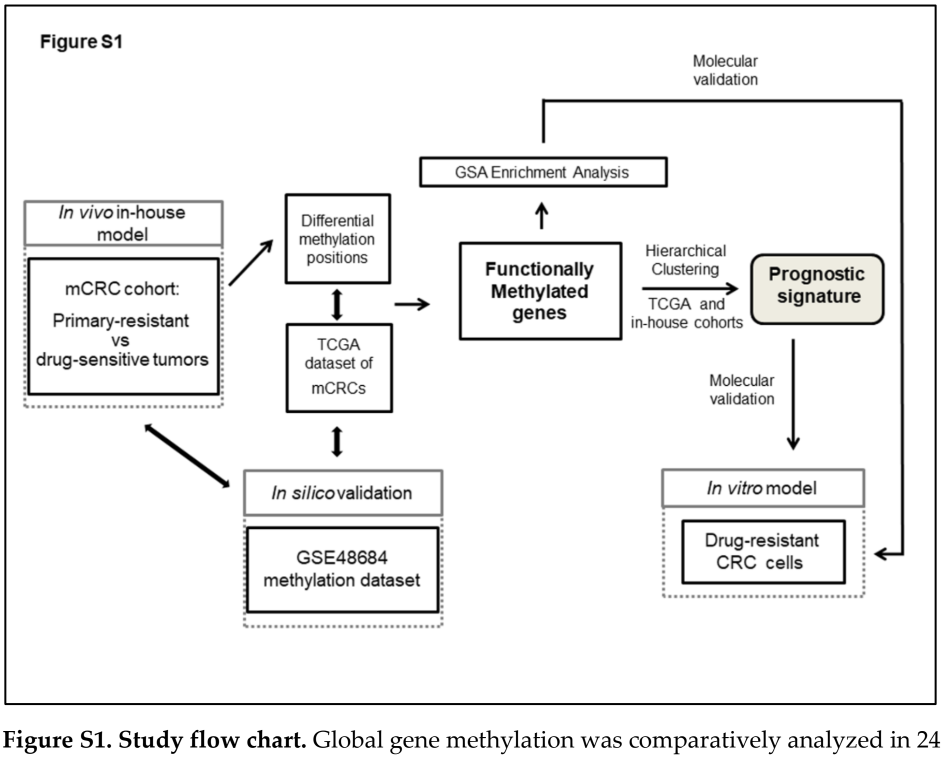

Figure S1. Study flow chart. Global gene methylation was comparatively analyzed in 24 first-line FOLFOX or FOLFIRI (± molecular-targeted agent) primary-resistant and 12 drug-sensitive mCRCs to obtain the differential methylation profile between two mCRC cohorts with significantly different outcome. Gene expression and methylation data of 33 mCRCs from The Cancer Genome Atlas

COlon ADenocarcinoma (TCGA COAD) database were used to restrict the list of Differentially Methylated Genes (DMGs) to those that are functionally methylated. Hierarchical clustering analysis on the TCGA COAD and the in-house cohorts was used to define the prognostic relevance of functionally methylated (fMET) genes (8-genes signature). GSA analysis was performed to identify significant enrichments for the positional and the signaling pathways collections. Methylation data from the GEO GSE 48684 dataset (24 mCRC patients) were used to further validate the list of DMGs and the enrichments obtained by GSA. Finally, the expression of statistically significant genes was validated in two independent oxaliplatin- and irinotecan-resistant CRC cell lines. GSE48684 dataset used to validate the in-house and TCGA methylation results.

Figure S2

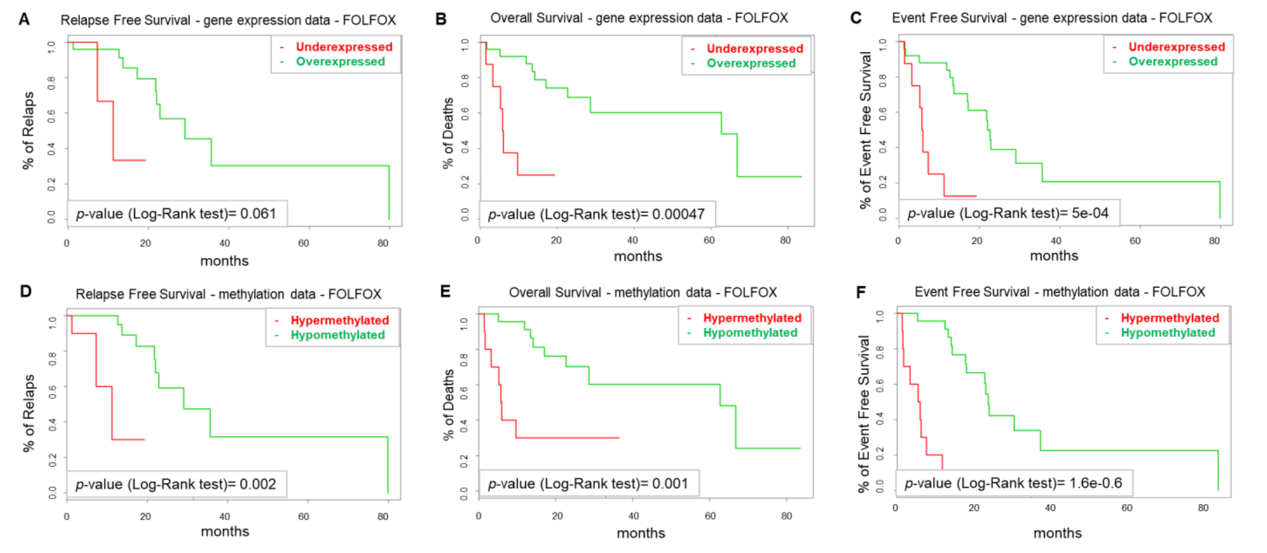

Figure S2. Kaplan-Meyer survival curves of mCRCs clusters according to the 5-genes 1st-line FOLFOX signature in TCGA COAD dataset. **A**–**F**. Relapse Free (**A** and **D**), Overall (**B** and **E**) and Event Free survival curves according to TCGA COAD hierarchical clusters reported in Figure 2A-B. Clusters were obtained according to differential expression (**A**, **B** and **C**) or methylation (**D**, **E** and **F**) data of the 1st-line FOLFOX 5-genes signature.

Figure S3

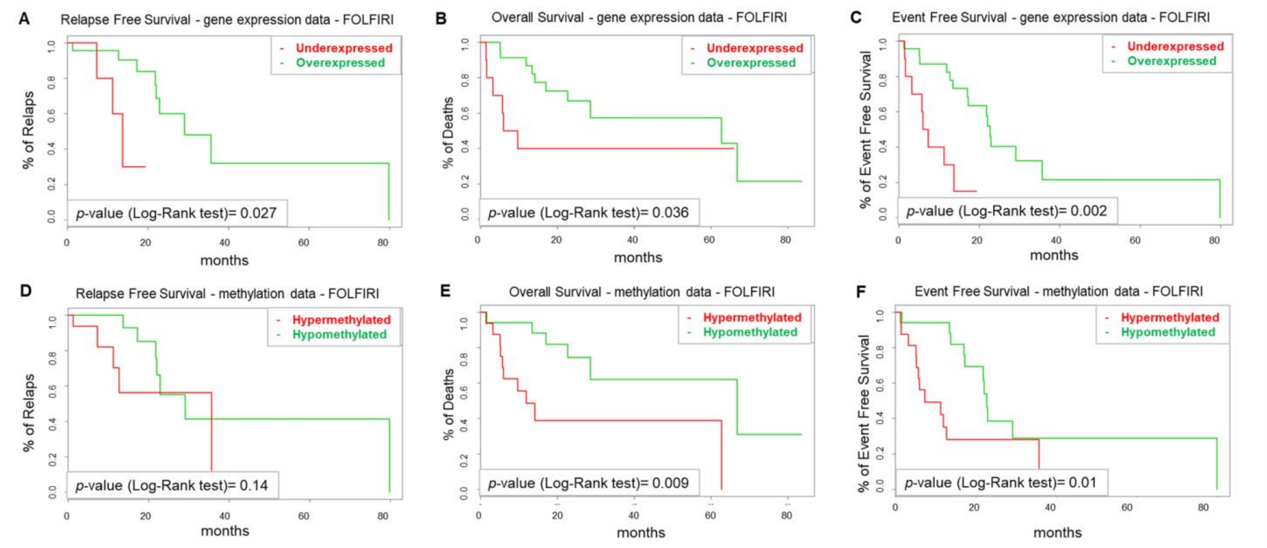

Figure S3. Kaplan-Meyer survival curves of mCRCs clusters according to the 4-genes 1st-line FOLFIRI signature in TCGA COAD dataset. **A**–**F**. Relapse Free (**A** and **D**), Overall (**B** and **E**) and Event Free survival curves according to

TCGA COAD hierarchical clusters reported in Figure 2D-E. Clusters were obtained according to differential expression (**A**, **B** and **C**) or methylation (**D**, **E** and **F**) data of the 1st-line FOLFIRI 4-genes signature.

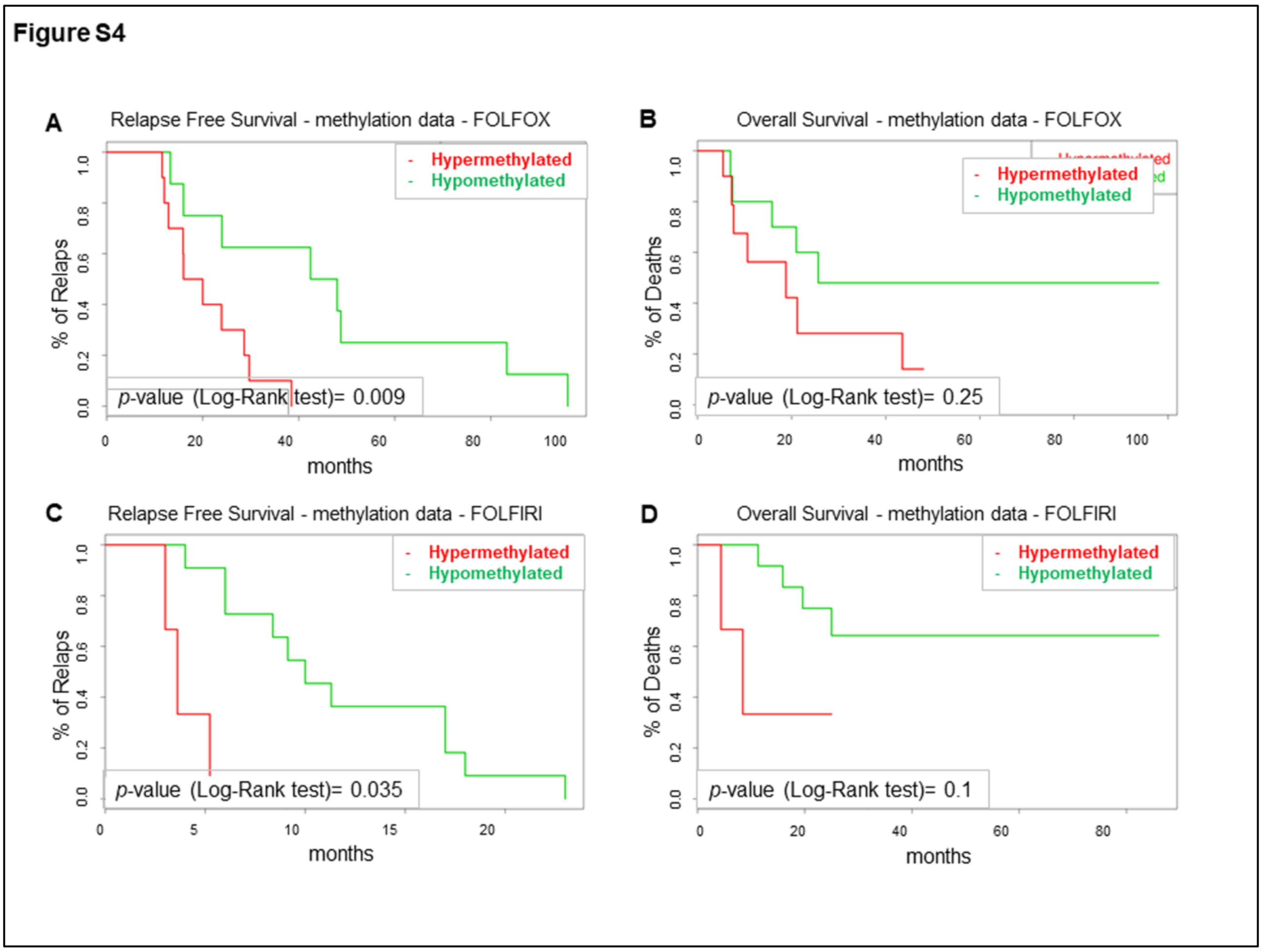

Figure S4. Kaplan-Meyer survival curves of mCRCs clusters according to the 5- and 4-genes signatures in 1st-line FOLFOX and FOLFIRI in-house datasets. **A**–**D**. Relapse Free (**A** and **C**) and Overall (**B** and **D**) survival curves according to hierarchical clusters reported in Figure 2C and F. Clusters were obtained according to methylation data of the 1st-line FOLFOX 5-genes (**A** and **B**) and 1st-line FOLFIRI 4-genes (**C** and **D**) signatures.

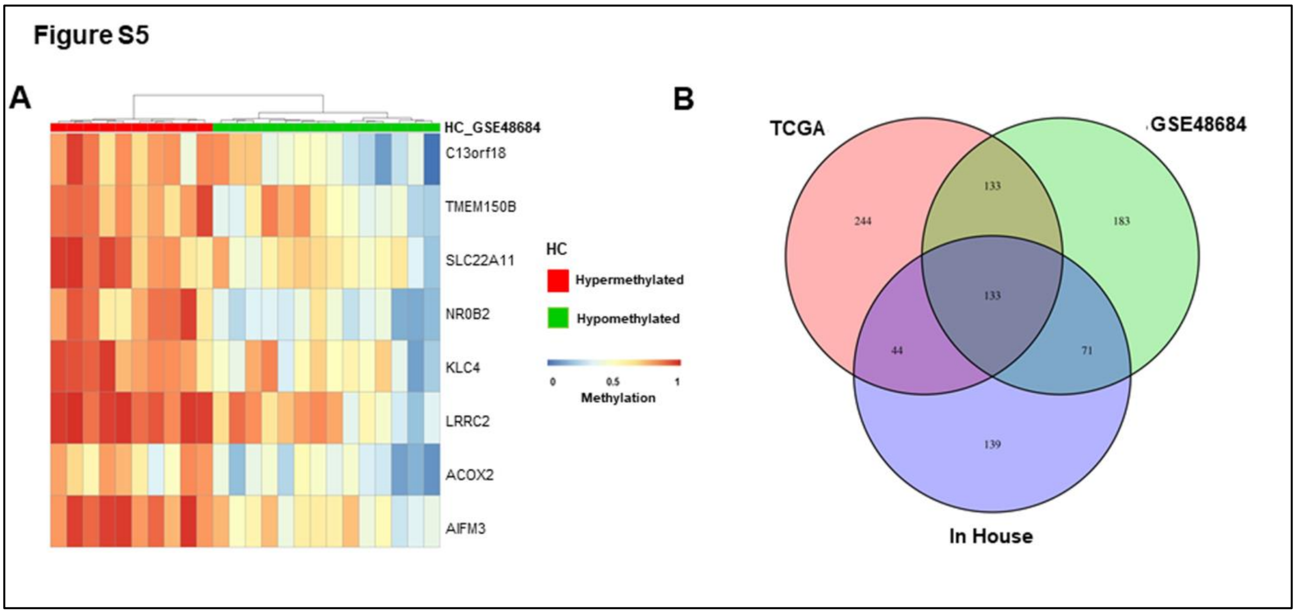

Figure S5. Comparative validation analysis in GSE48684, TCGA and whole in-house datasets. A. Heatmap (HM) of the 8-genes signature in 24 mCRCs from GSE48684 dataset. B. Venn diagram of the Pathways enriched in the three datasets. HC, hierarchical clustering.

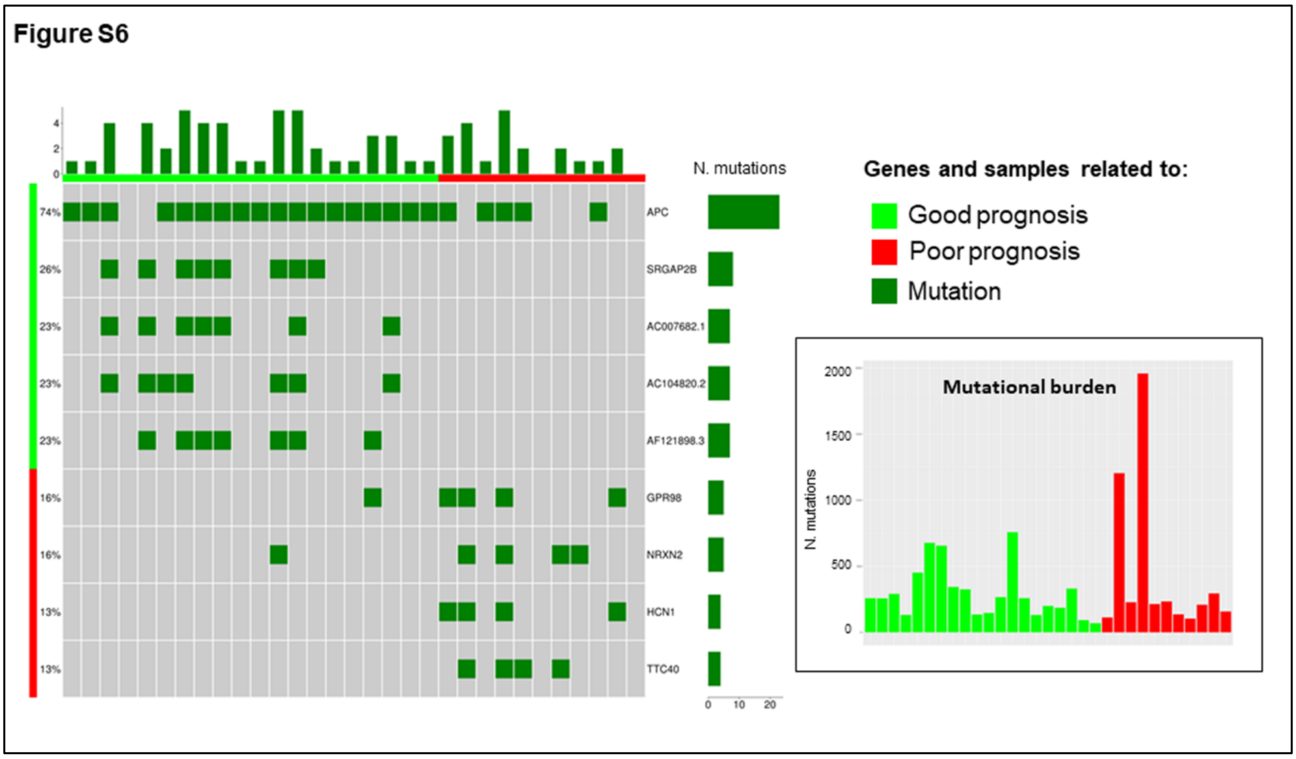

Figure S6. DNA mutational characterization of the poor and good prognosis clusters. HM of gene mutations differentially distributed (Fisher test, p-value < 0.05) between the good and the poor prognosis clusters. Insert: mutational burden in TCGA-COAD samples.

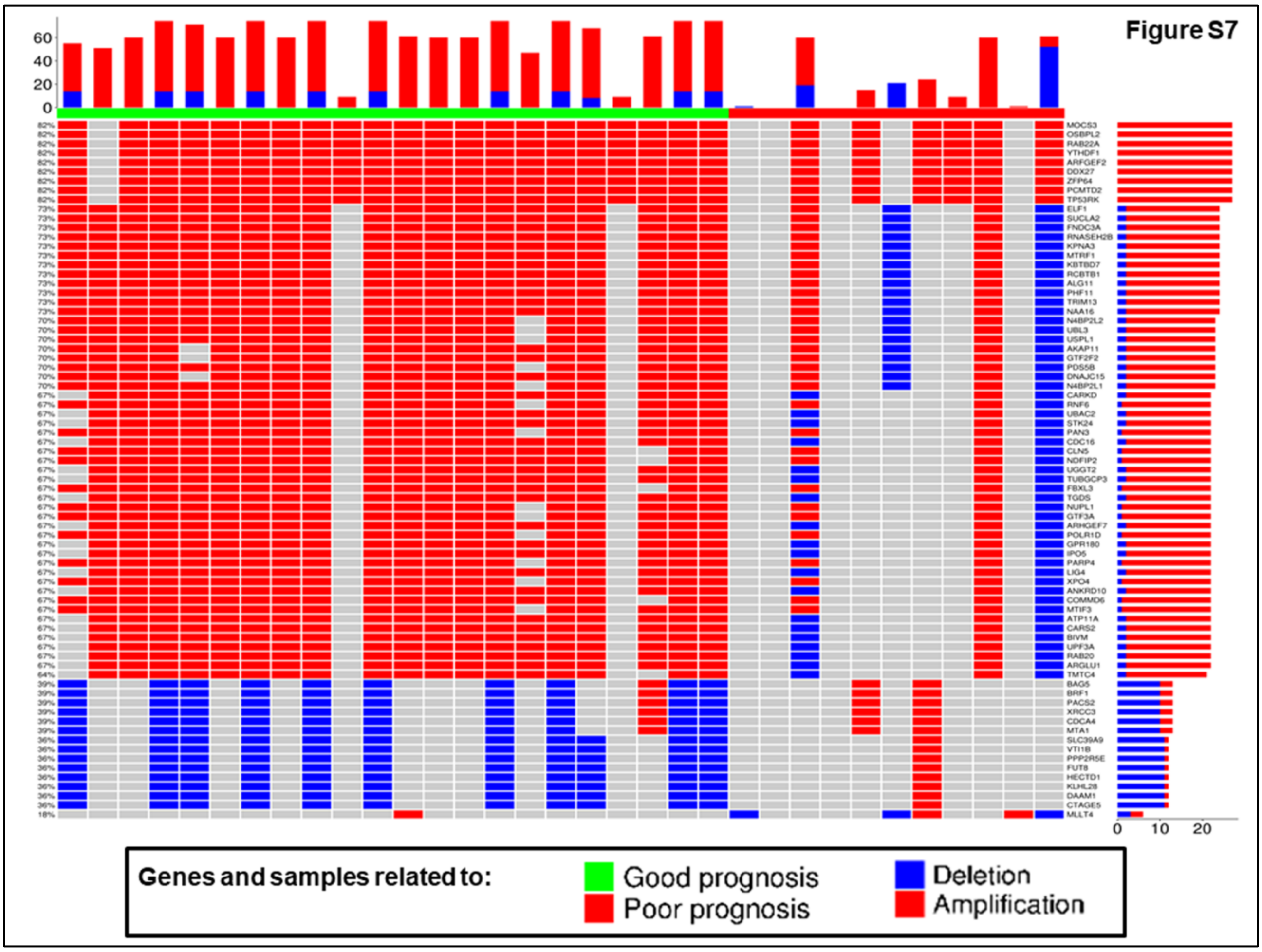

Figure S7. Gene aberrations in poor and good prognosis clusters. Aberrations of functionally selected genes (genes with $R^2 > 0.5$ between expression and copy number) in TCGA-COAD samples. The HM reports genes with significant (Fisher test, p-value < 0.01) different aberrations abundance between the good and the poor prognosis clusters.

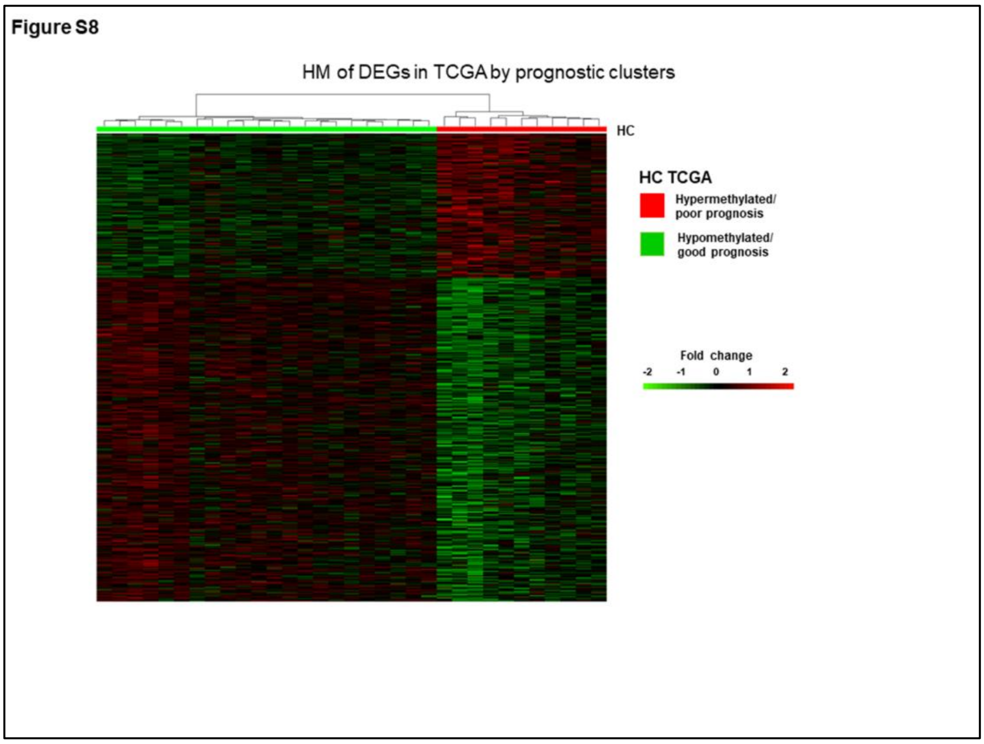

Figure S8. Heatmap (HM) of 444 Differentially Expressed Genes (DEGs) between the TCGA hypermethylated/poor prognosis and hypomethylated/good prognostic clusters. HC, hierarchical clustering.

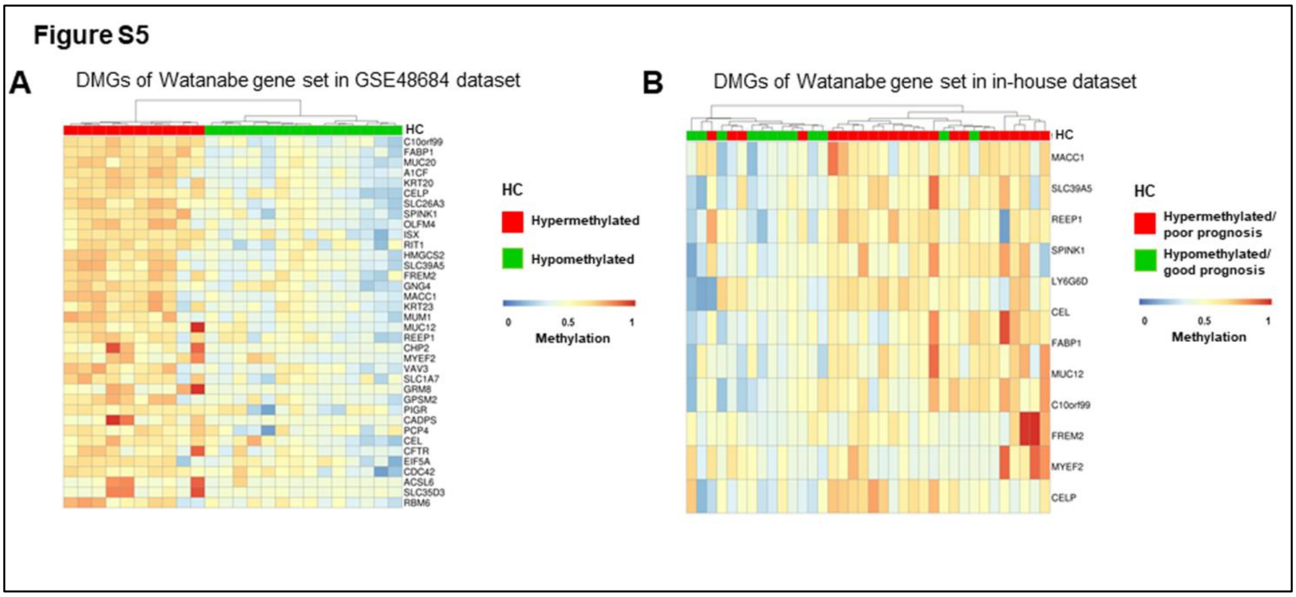

Figure S9. Watanabe signature in GSE48684 and in-house datasets. A–B. HMs of differentially methylated probes enriching Watanabe gene set in GSE48684 (**A**) and in-house (**B**) datasets. HC, hierarchical clustering.

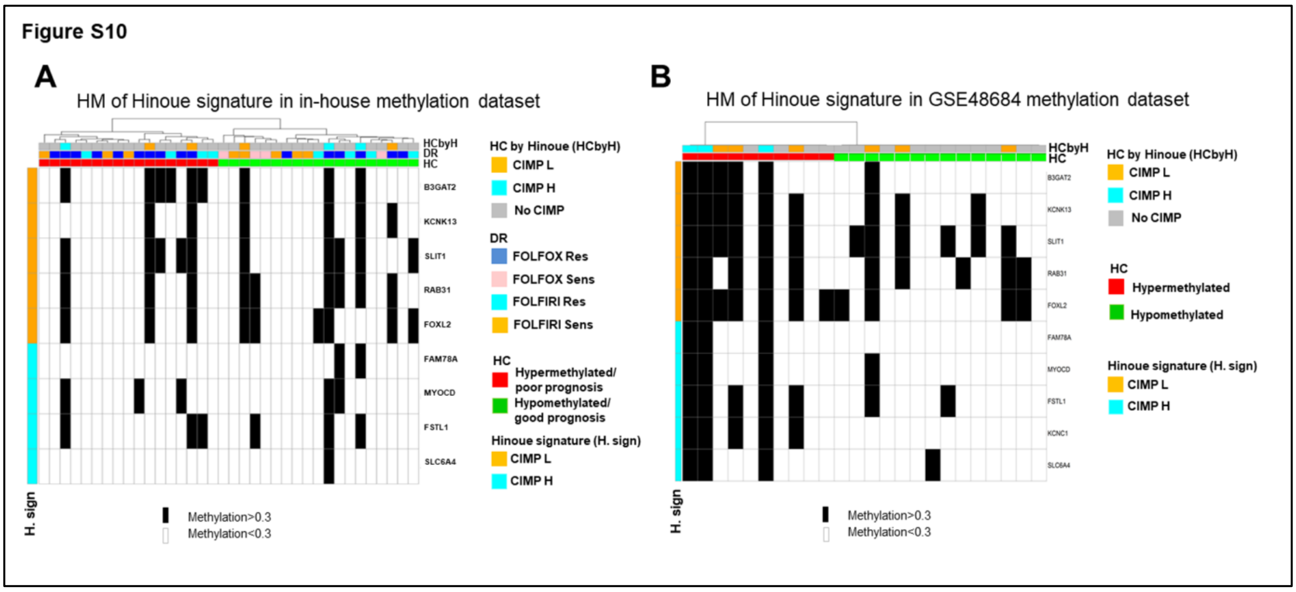

Figure S10. CIMP status in in-house and GSE48684 datasets. **A–B** Heatmap (HM) of CIMP status in mCRCs from in-house (**A**) and GSE48684 (**B**) datasets according to the 8-genes signature. CIMP status is labeled in black.

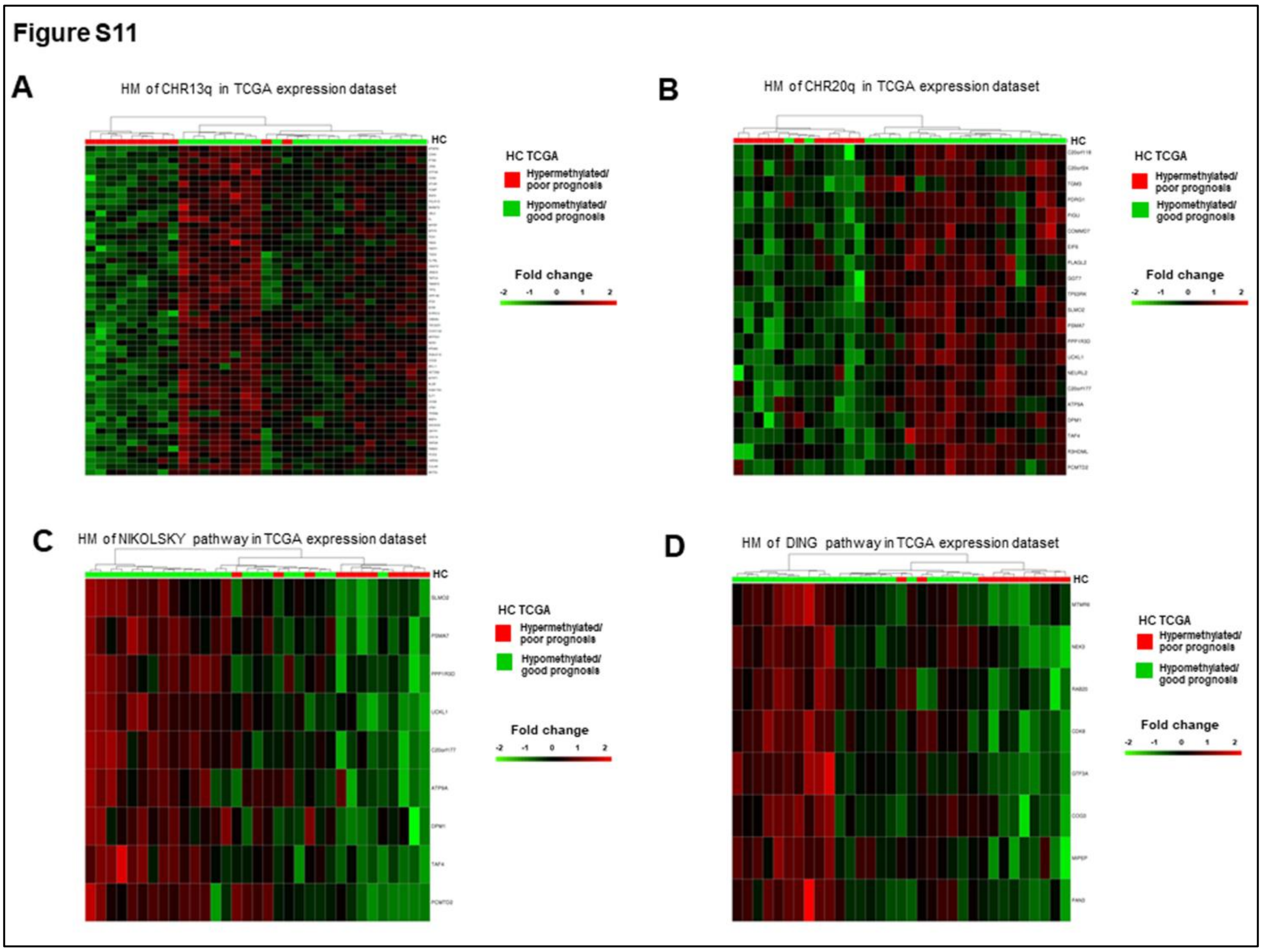

Figure S11. Hierarchical clustering according to enrichment analysis gene sets. A–B. Heatmaps (HM) of differentially expressed genes enriching chromosome 13 arm q (**A**) and chromosome 20 arm q (**B**) gene sets in 33 mCRCs from TCGA COAD dataset. **C–D**. Heatmaps of differentially expressed genes enriching Nikolsky (**C**) and Ding (**D**) gene sets in 33 mCRCs from COAD TCGA dataset. HC, hierarchical clustering.

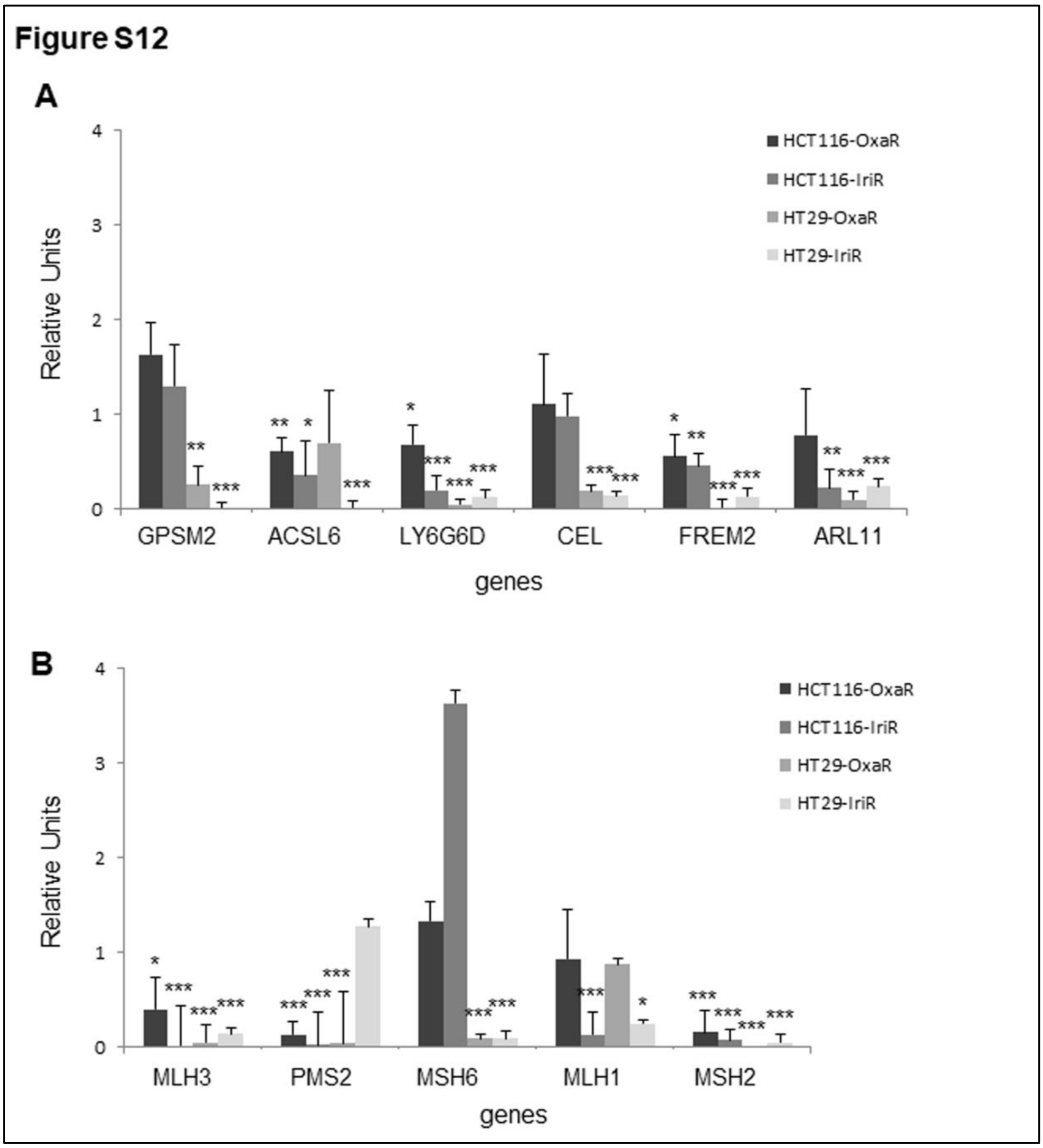

Figure S12. Validation of Watanabe and mismatch repair genes in drug-resistant CRC cell lines. A–B. Real time differential expression analysis of 7 genes enriching Watanabe gene set (**A**) and 5 genes of the mismatch repair system (**B**) between drug-resistant and drug-sensitive CRC cell lines before and after exposure to 10 uM 5-Aza-dC for 48h. Genes significantly modulated are indicated by asterisk: * = $p < 0.05$; ** = $p < 0.01$; *** = $p < 0.001$. PCR analysis was performed in triplicate.

Supplementary Tables

Table S1. Baseline patients' characteristics of in-house cohort.

		**Primary-resistant (*n* = 24)**	**Drug-sensitive (*n* = 12)**	**Total (*n* = 36)**
Age	Range	37-76	29-81	29-81
	Median	65.5	58.5	61
Gender	Male	17	7	24
	Female	7	5	12
First-line Therapy	FOLFOX	10	-	10
	FOLFOX/ Bevaci-	4	3	7

	zumab	2	1	3
	FOLFOX/anti-EGFR	3	4	7
	FOLFIRI/ Bevaci-	5	4	9
	zumab			
	FOLFIRI/anti-EGFR			
Metastatic pattern	Liver	19	6	25
	Lung	10	5	15
	Peritoneum	8	1	9
	Others	15	3	18

Table S2. Baseline patients' characteristics of the TCGA cohort.

**TCGA cohort**		**Hypermethylated Poor prognosis (*n* = 11)**	**Hypomethylated Good prognosis (*n* = 22)**	**Total (*n* = 33)**
Age	Range	45–71	54–76	52–72
	Median	68.3	59.3	61.4
Gender	Male	6	13	19
	Female	5	9	14
TNM	T1	/	/	/
	T2	/	/	/
	T3	6	16	22
	T4	2	2	4
	T4a	2	3	5
	T4b	1	1	2
	N0	2	3	5
	N1	2	12	14
	N2	7	7	14
Site of primary tumor	Right	7	8	15
	Left	4	14	18

Table S3. Functionally methylated genes obtained from the intersection of in-house and TCGA datasets.

**Entrez**	**CpG**	**HCGN gene symbol**	**Gene**	**Dataset**
89953	cg16370701	*KLC4*	Kinesin Light Chain 4	FOLFOX
79442	cg08058988	*LRRC2*	Leucine rich repeat containing 2	FOLFOX
8309	cg16209444	*ACOX2*	Acyl-CoA oxidase 2	FOLFOX
150209	cg00280270	*AIFM3*	Apoptosis Inducing Factor Mitocondria Associated 3	FOLFOX
80183	cg08879684	*RUBCNL(C13orf18)*	Rubicone like autophagy enhancer	FOLFOX/FOLFIRI
284417	cg04391588	*TMEM150B*	Transmembrane Protein 150B	FOLFIRI
8431	cg06650260	*NR0B2*	Nuclear Receptor superfamily O group B member 2	FOLFIRI
55867	cg09326702	*SLC22A11*	Solute carrier family 22 member 11	FOLFIRI

Table S4. Oligonucleotides utilized in Real Time RT-PCR analysis.

Primer name	Sequence (5′-3′)	Amplicone size
KLC4 For<br>KLC4 Rev	ATGGACATACCTCGGAGG<br>TGCTGTAGCACCTTGACC	122 bp
LRRC2 For<br>LRRC2 Rev	AAGAATGGCTTCATAGACACC<br>TGTACCATTCTCTCAGGTGTG	190 bp
ACOX2 For<br>ACOX2 Rev	ACTTCAGCTTCCTGCCTG<br>AGGTAGAGCACTGTGTTCTCAC	199 bp
AIFM3 For<br>AIFM3 Rev	TACACTAAAGGCGACGAGG<br>AGCTCAGGATCCTTTCCC	156 bp
C13orf18 For<br>C13orf18 Rev	AGAGTGTCACTTATGAGCCAGAC<br>TTCGGACACACTCTTCATTTAC	162 bp
TMEM150B For<br>TMEM150B Rev	TAGCTGTCTGGGCTATCTCTG<br>ATCCGCAGATGCTGATG	105 bp
NR0B2 For<br>NR0B2 Rev	TGGAGATGTTGACATCGC<br>AGGAGCCAAGTGCTGTCTATAC	177 bp
SLC22A11 For<br>SLC22A11 Rev	ATTCTAGCCAACATGCTGG<br>TGGAAAGAGTTCAGCCTTG	126 bp
GPSM2 For<br>GPSM2 Rev	AATCGGCAGACCATTAGTTAC<br>TGTCCAGAGAAGTATTAATGCC	186 bp
WIF1 For<br>WIF1 Rev	ACGATGTATGAATGGTGGAC<br>ATTTCACACTGCTCTCCCTC	177 bp
ACSL6 For<br>ACSL6 Rev	AAGGTTATGGCCAAACTGAG<br>TGAACACATTTGGTCCTCTC	183 bp
LY6G6D For<br>LY6G6D Rev	AGTTCTTGCAAAGAGGCC<br>TCTCCACTTGATTGCAATG	148 bp
CEL For<br>CEL Rev	AACAACTACCTGTATGACGGC<br>TTACCTGGCAGATTGGC	122 bp
FREM2 For<br>FREM2 Rev	TTCTGAAGACTCTGACCAGG<br>TGACCCTCATAGAGAATAAGACC	165 bp
ARL11 For<br>ARL11 Rev	ACGTGTCACTGACTCTCTGG<br>TCGTTCAGGACTTCTGTGAG	160 bp
MLH3 For<br>MLH3 Rev	TCTCAGAATGGGACAATCC<br>TACTTGCTGGAGAACCTGC	161 bp
PMS2 For<br>PMS2 Rev	AGGAATATTAAGAAGGAGTATGCC<br>AGCCGATATTTTCCTTTATGC	163 bp
MSH6 For<br>MSH6 Rev	TTAATGCAGCAAGGCTTG<br>TTATGGACAGCTTCAGCATC	157 bp
MLH1 For<br>MLH1 Rev	AGCAGTACATATCTGAGGAGTCG<br>AGGCAGGTTAGCAAGCTG	179 bp
MSH2 For<br>MSH2 Rev	TCACCACTGAAGAGACCTTAAC<br>TGATATCATATCCTTGCGATTC	183 bp
Beta-actin For<br>Beta-actin Rev	CGCAAAGACCTGTACGC<br>CACACGGAGTACTTGCGC	152 bp

bp, base pair.

Supplementary Datasets

Dataset S1. Differentially methylated promoter probes between primary-resistant and drug-sensitive mCRCs

Dataset S2. Differentially methylated genes (DMGs) between drug-resistant and drug-sensitive tumors after TCGA intersection.

Dataset S3. COAD fMET genes

Dataset S4. Overlap between the DMGs obtained in the three datasets

Dataset 5. Overlap between the mSigDB gene sets enriched in in-house and GSE48684

Dataset S6. Overlap between the mSigDB gene sets enriched in in-house and TCGA

Dataset S7. Overlap between the mSigDB gene sets enriched in TCGA and GSE48684

Dataset S8. Overlap between the mSigDB gene sets enriched in TCGA expression samples using methylation or expression based clustering
