# Supplementary figures and images for: Novel Epigenetic Eight-Gene Signature Predictive of Poor Prognosis and MSI-Like Phenotype in Human Metastatic Colorectal Carcinomas
Source: Cancers (Basel). 2021 Jan 5;13(1):158. doi: 10.3390/cancers13010158 (PMC7796477; doi:10.3390/cancers13010158)

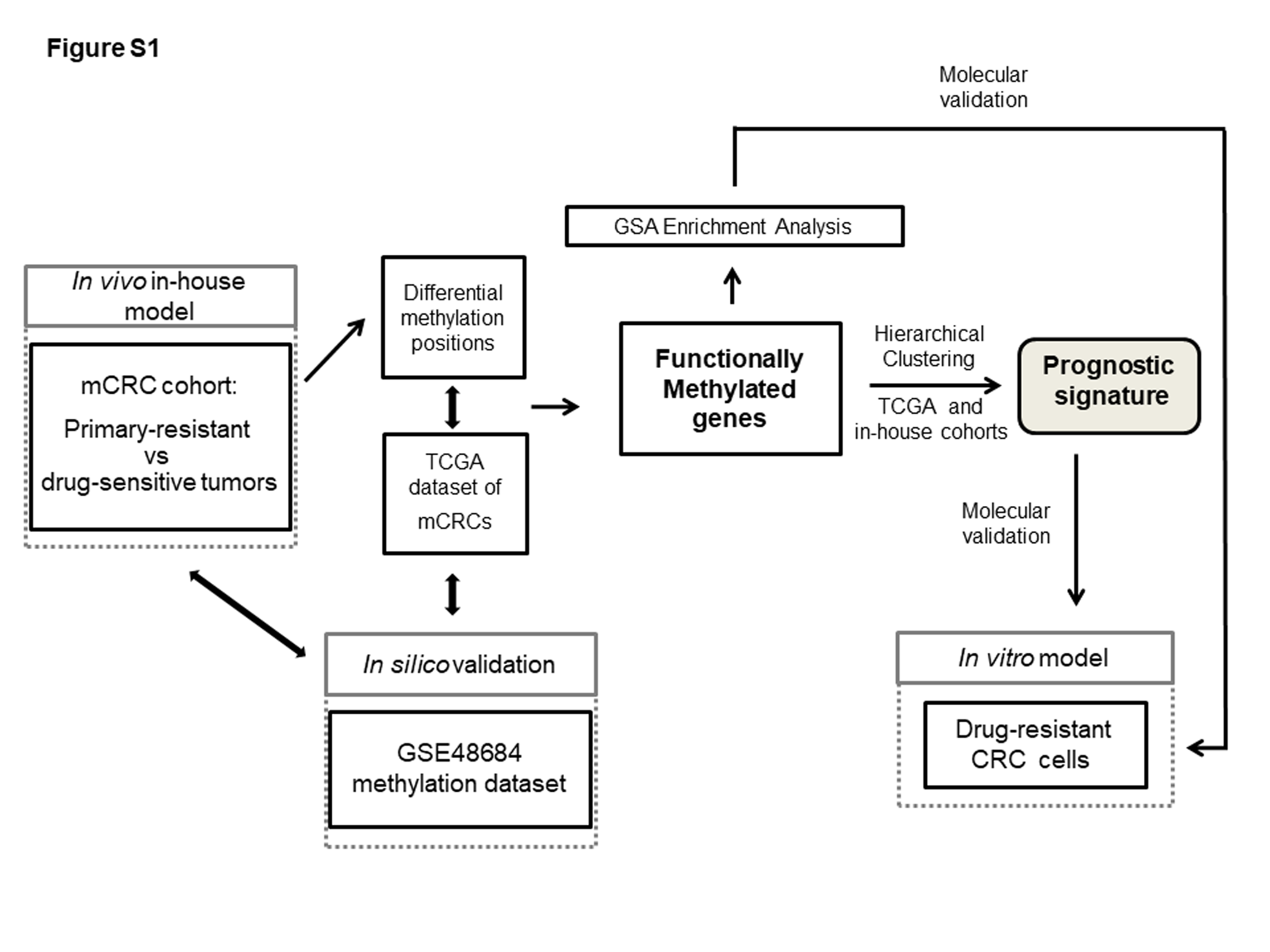

Supplement: Supplementary file 1 [file cancers-13-00158-s001.zip › Supplementary information/Supplementary Figures/Supplementary Figure S1.tif]

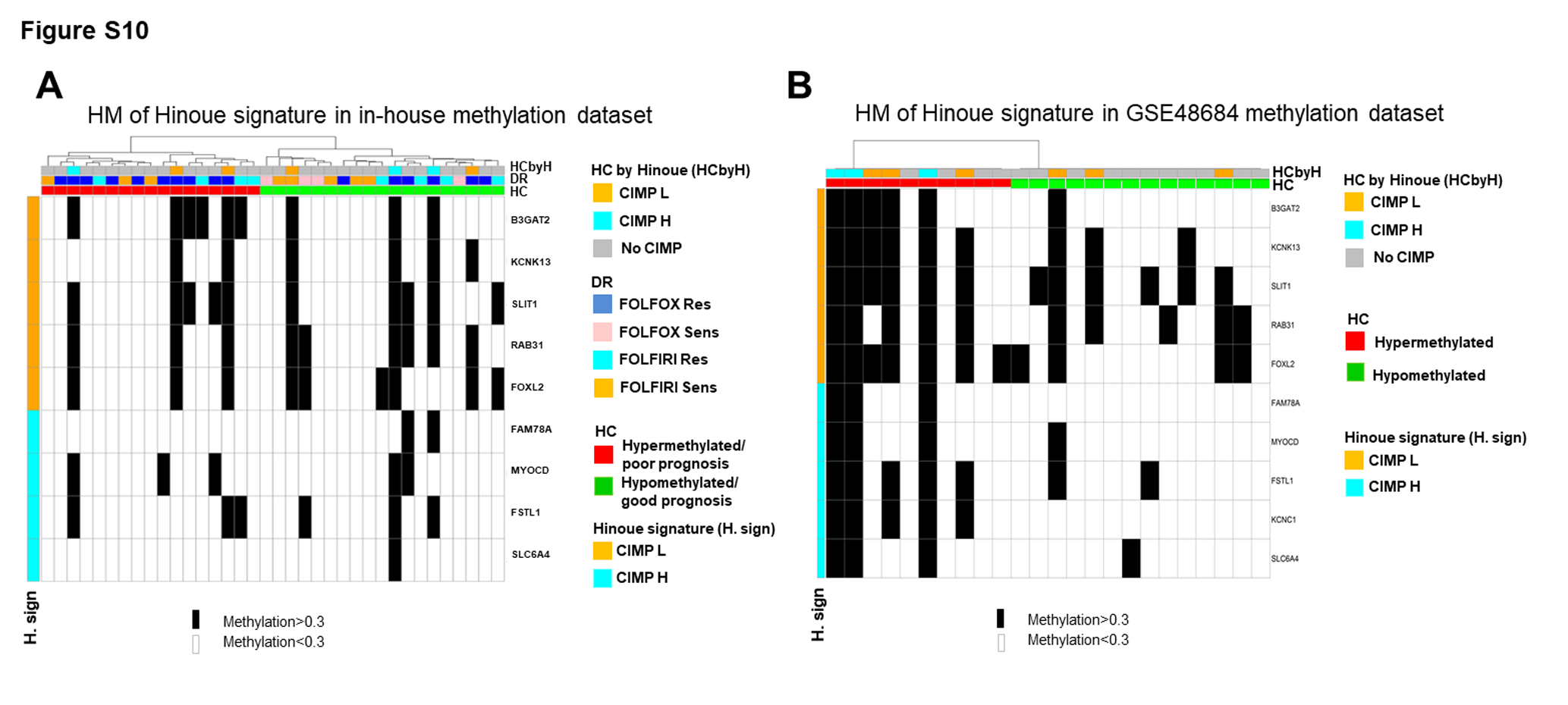

Supplement: Supplementary file 1 [file cancers-13-00158-s001.zip › Supplementary information/Supplementary Figures/Supplementary Figure S10.tif]

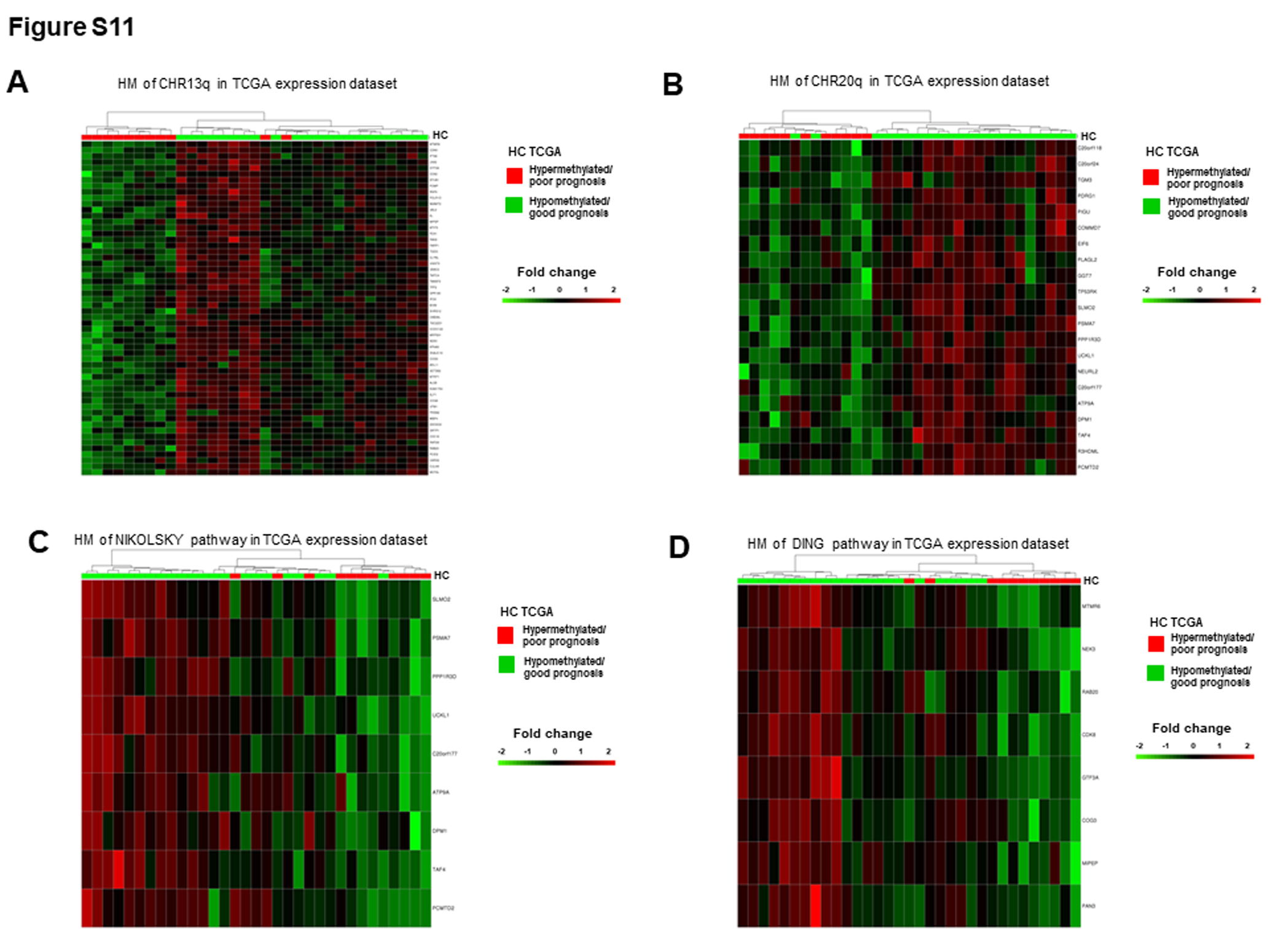

Supplement: Supplementary file 1 [file cancers-13-00158-s001.zip › Supplementary information/Supplementary Figures/Supplementary Figure S11.tif]

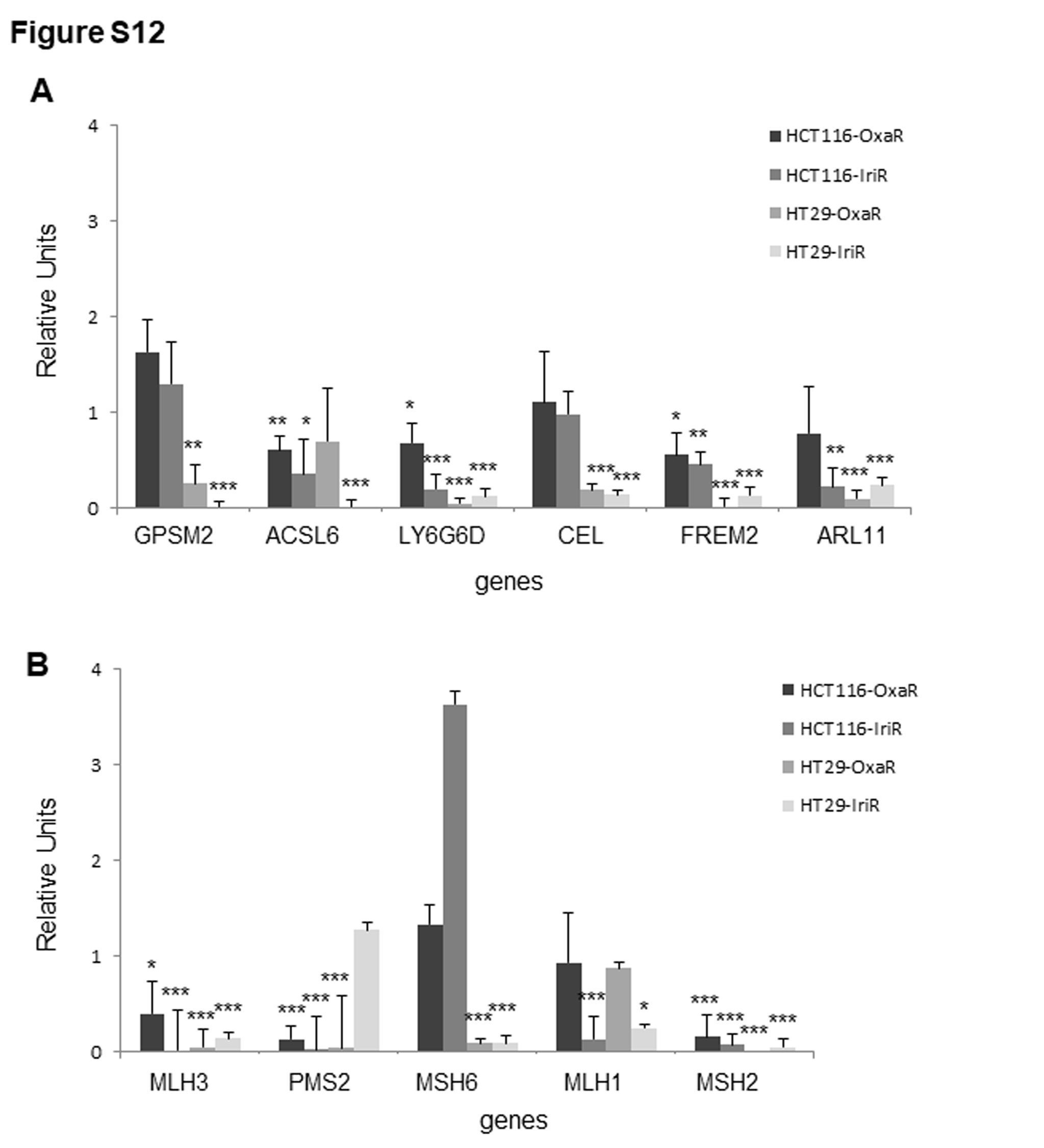

Supplement: Supplementary file 1 [file cancers-13-00158-s001.zip › Supplementary information/Supplementary Figures/Supplementary Figure S12.tif]

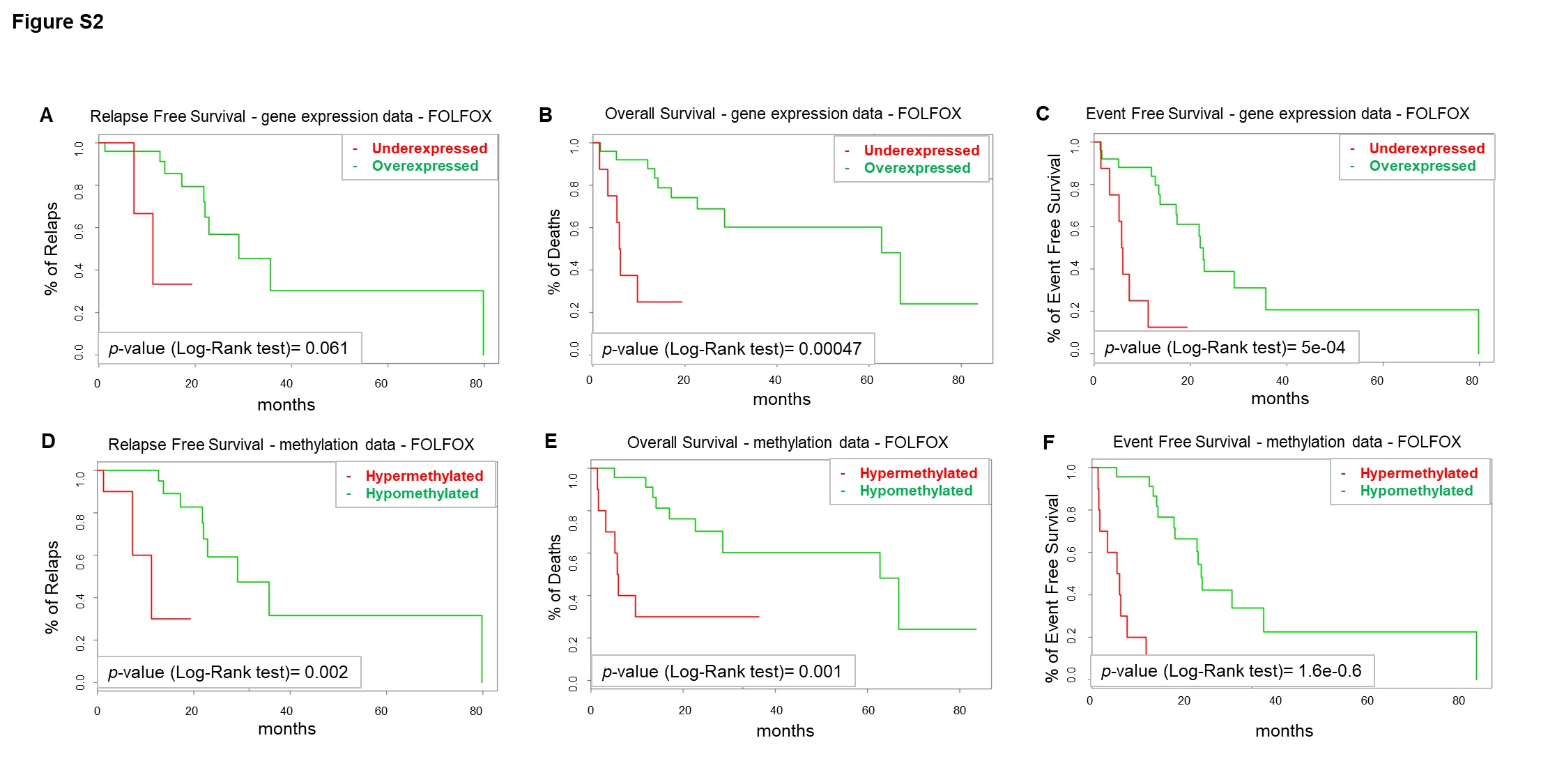

Supplement: Supplementary file 1 [file cancers-13-00158-s001.zip › Supplementary information/Supplementary Figures/Supplementary Figure S2.tif]

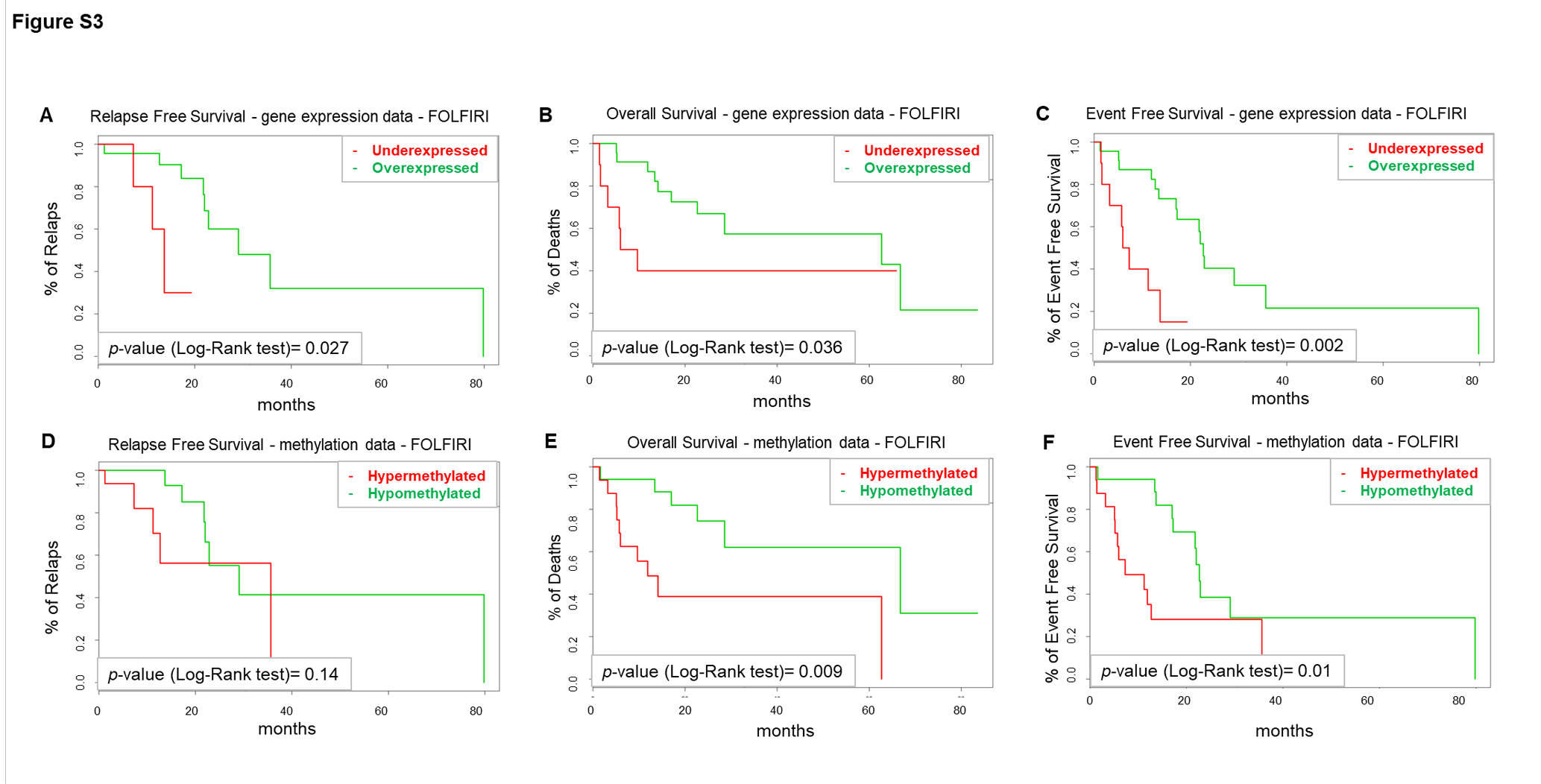

Supplement: Supplementary file 1 [file cancers-13-00158-s001.zip › Supplementary information/Supplementary Figures/Supplementary Figure S3.tif]

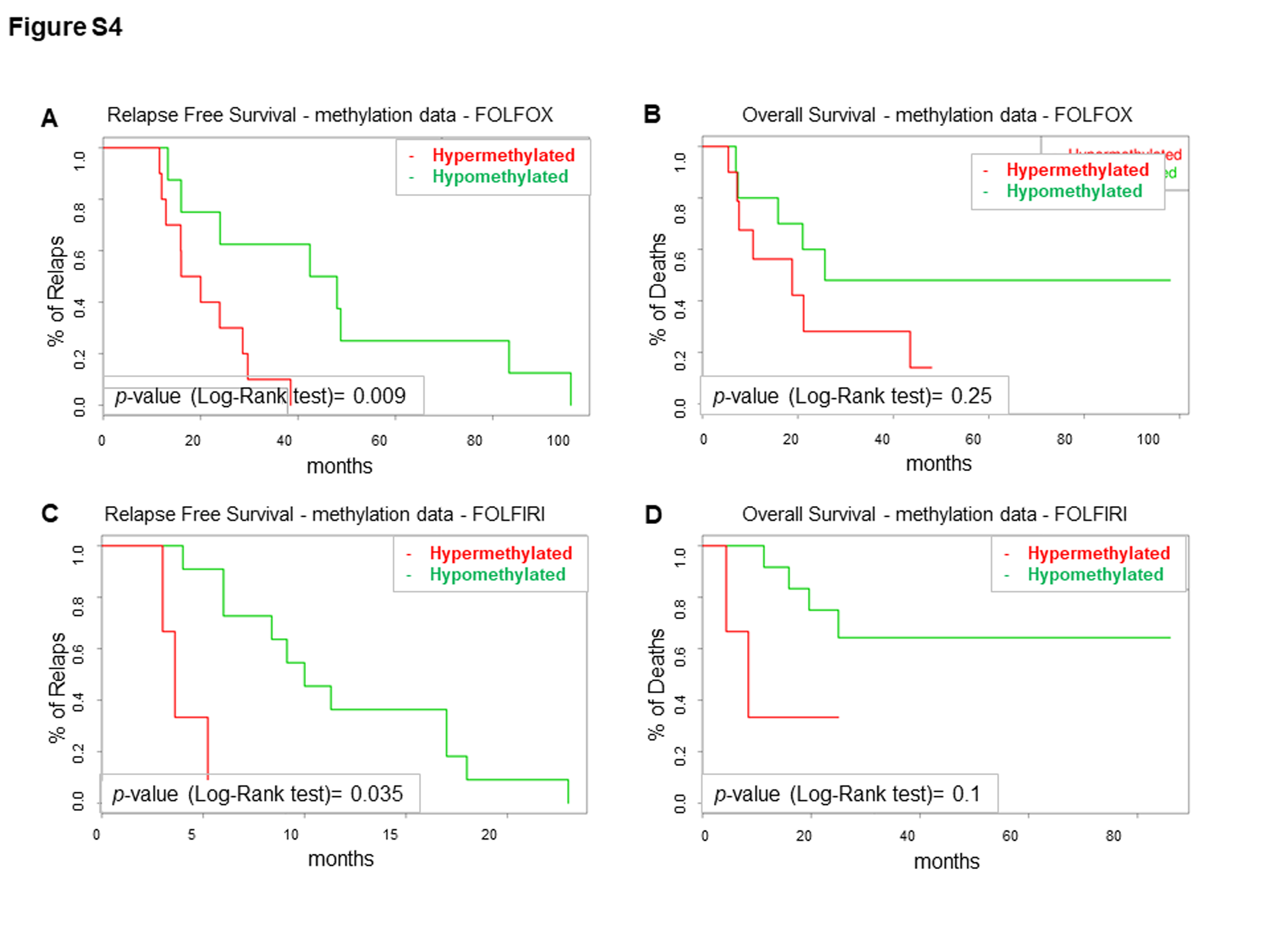

Supplement: Supplementary file 1 [file cancers-13-00158-s001.zip › Supplementary information/Supplementary Figures/Supplementary Figure S4.tif]

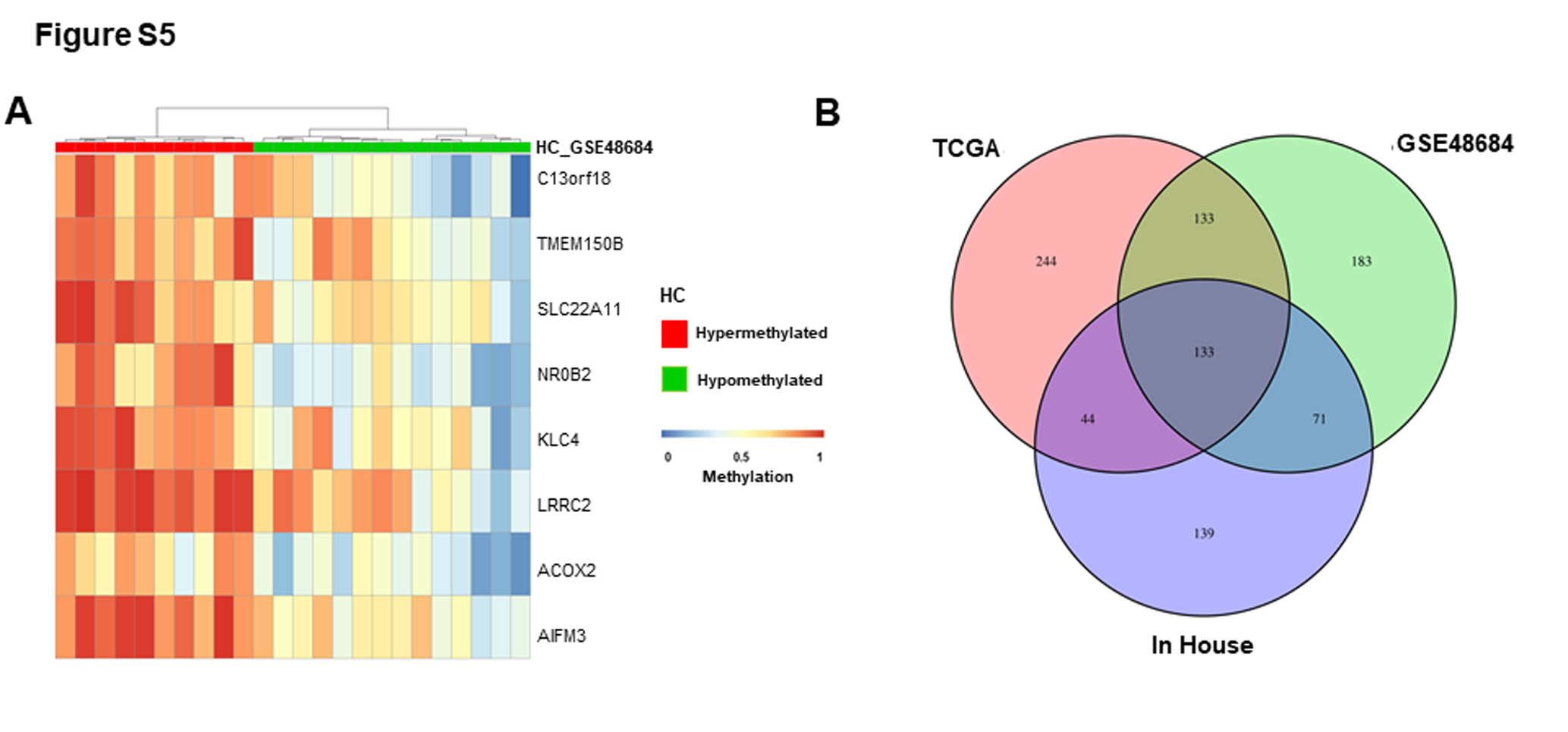

Supplement: Supplementary file 1 [file cancers-13-00158-s001.zip › Supplementary information/Supplementary Figures/Supplementary Figure S5.tif]

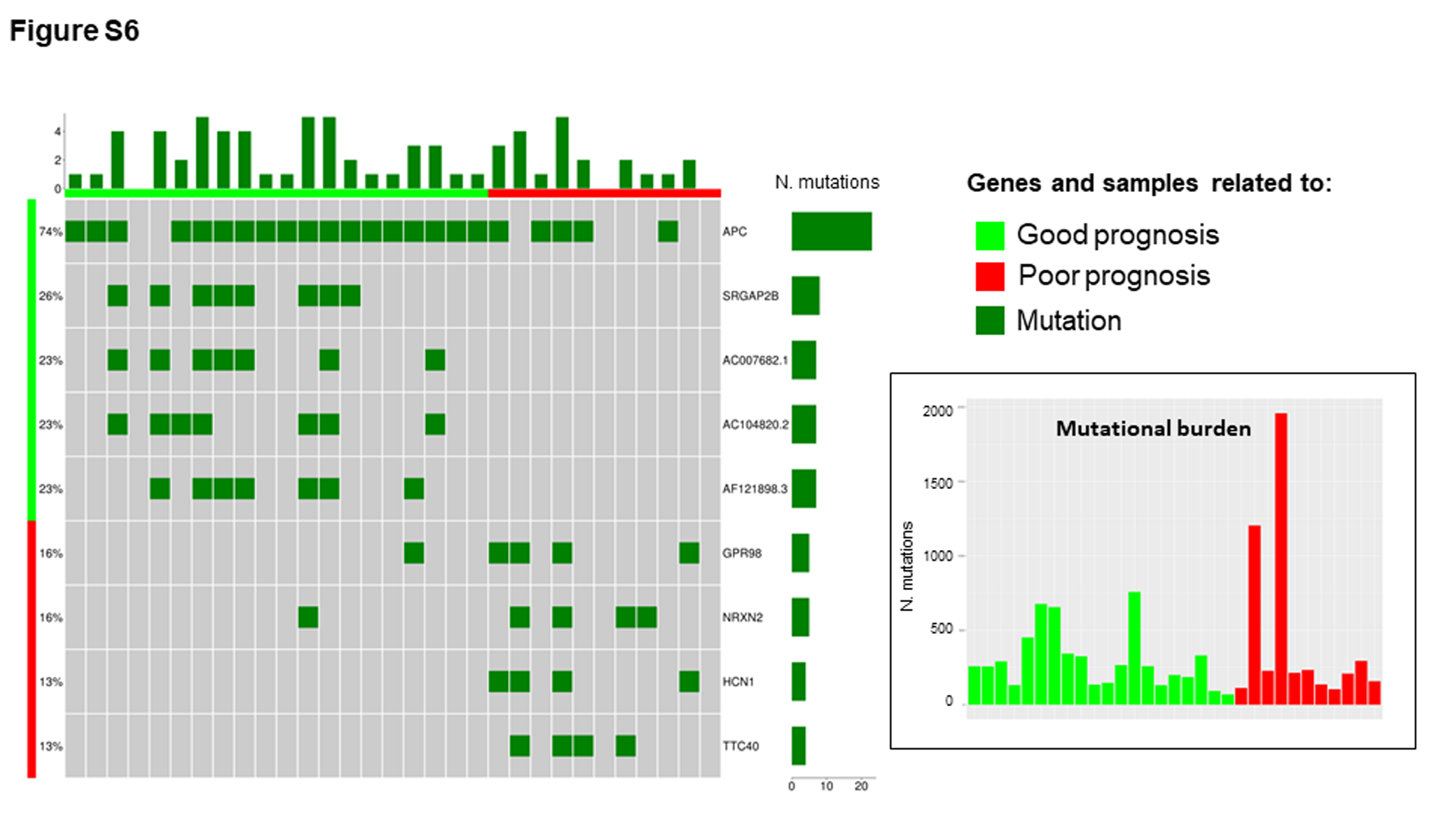

Supplement: Supplementary file 1 [file cancers-13-00158-s001.zip › Supplementary information/Supplementary Figures/Supplementary Figure S6.tif]

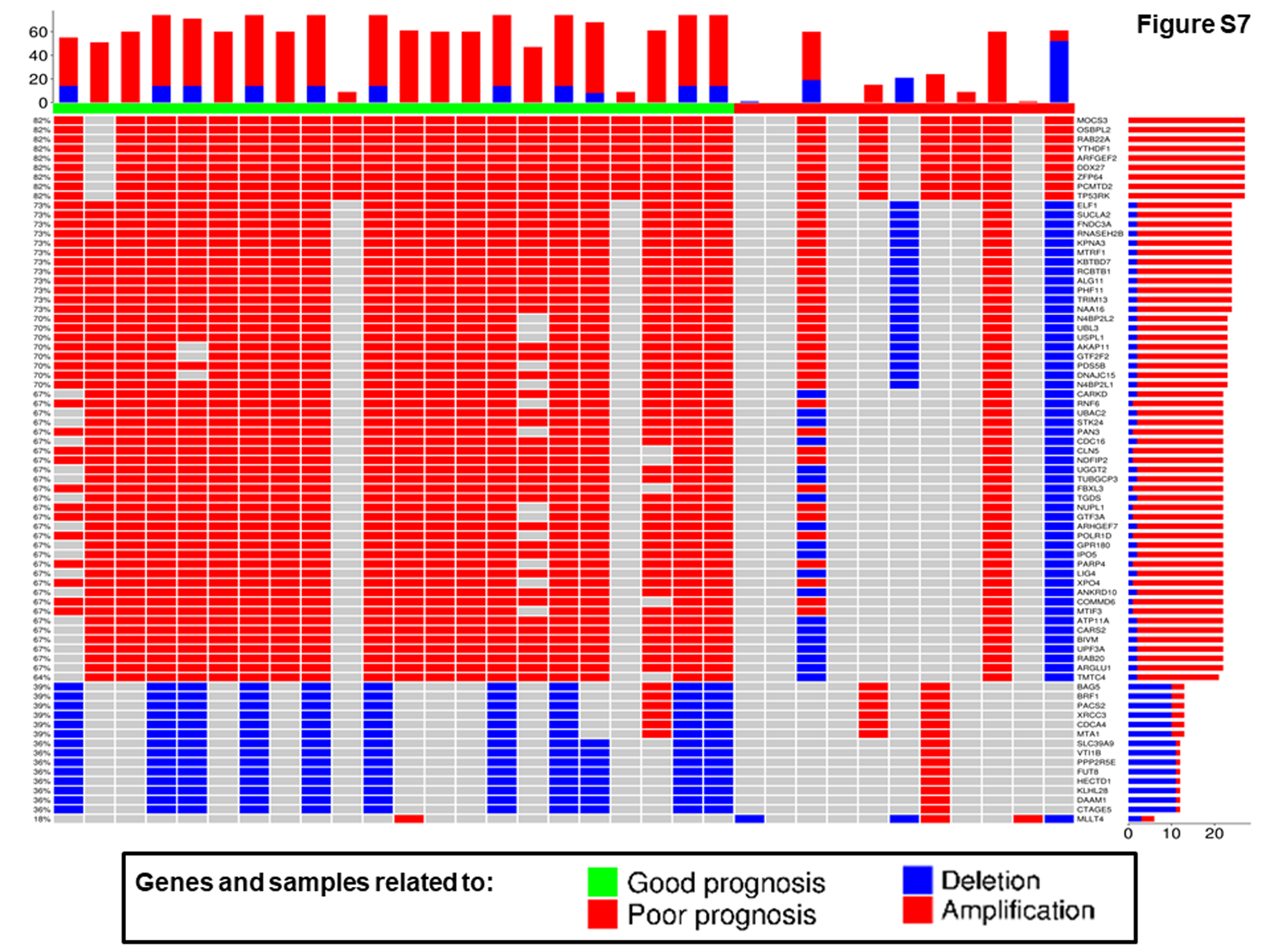

Supplement: Supplementary file 1 [file cancers-13-00158-s001.zip › Supplementary information/Supplementary Figures/Supplementary Figure S7.tif]

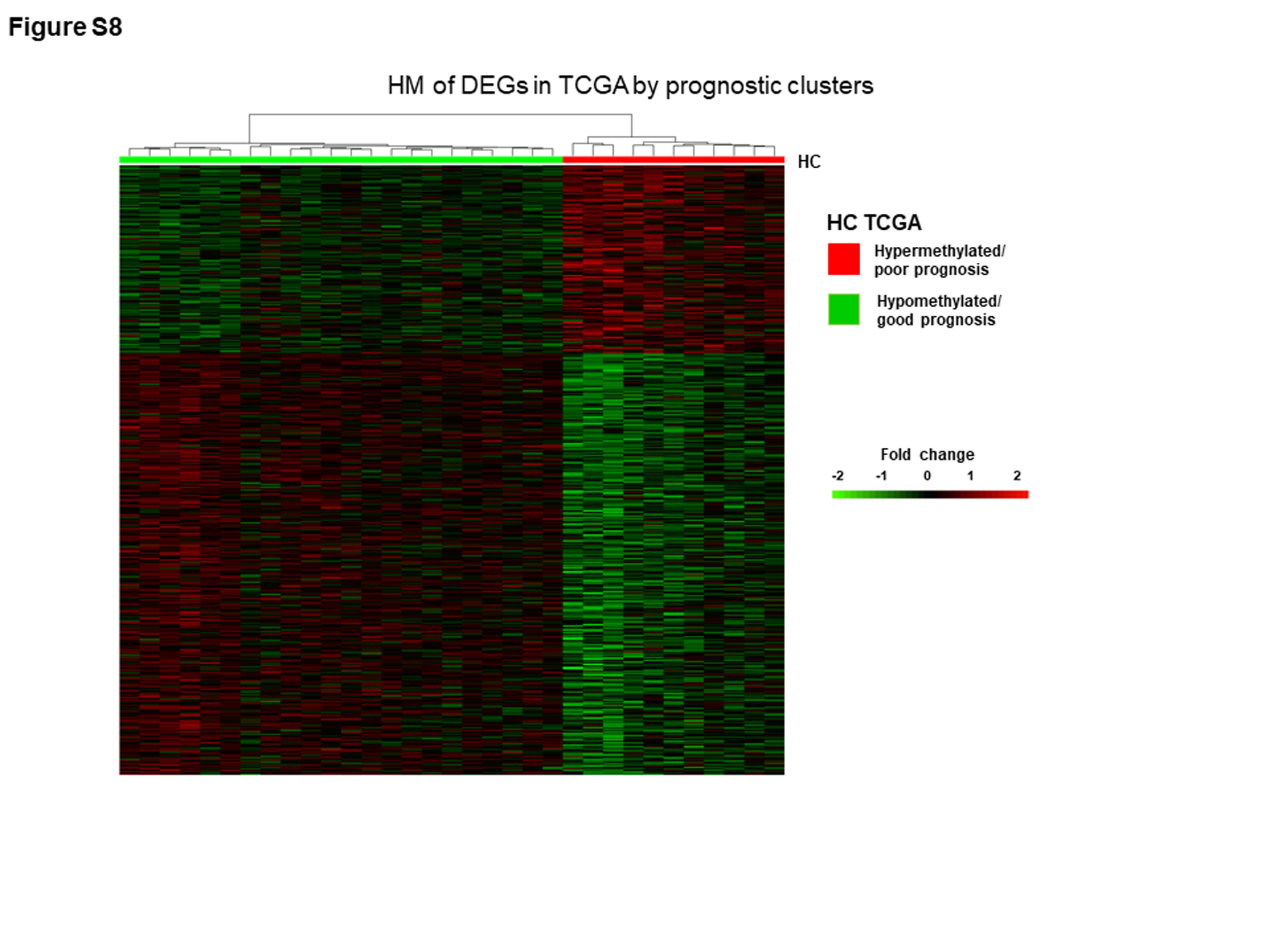

Supplement: Supplementary file 1 [file cancers-13-00158-s001.zip › Supplementary information/Supplementary Figures/Supplementary Figure S8.tif]

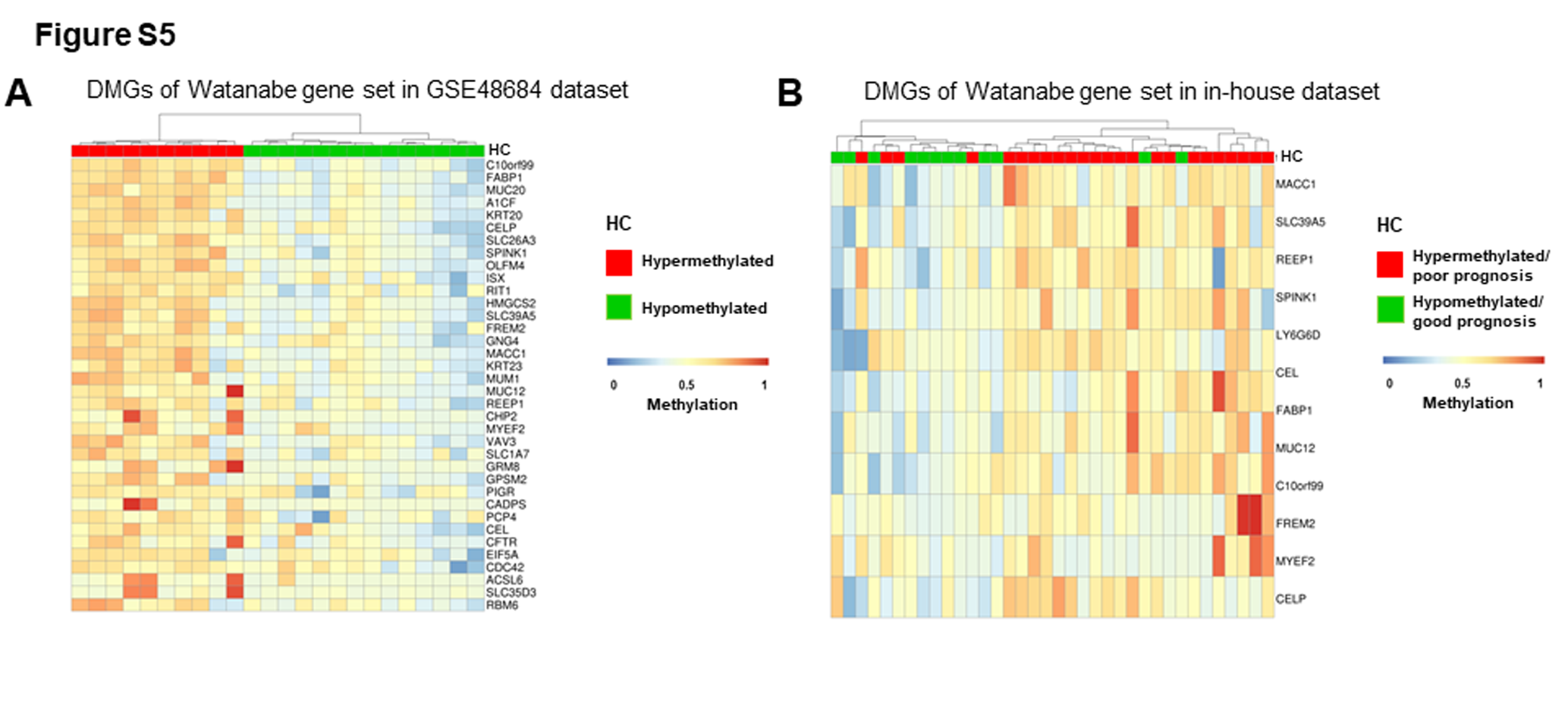

Supplement: Supplementary file 1 [file cancers-13-00158-s001.zip › Supplementary information/Supplementary Figures/Supplementary Figure S9.tif]
